# Effectiveness of SMS-based interventions in enhancing antenatal care in developing countries: a systematic review

**DOI:** 10.1136/bmjopen-2024-089671

**Published:** 2025-02-25

**Authors:** Mahamadou Kante, Mats Målqvist

**Affiliations:** 1 Department of Women's and Children’s Health, Uppsala University, Uppsala, Sweden; 2 Computer Science, Institut des Sciences Humaines, Bamako, Mali

**Keywords:** Health informatics, Information technology, Pregnant Women, PUBLIC HEALTH

## Abstract

**Abstract:**

**Objectives:**

Pregnant women in low- and middle-income countries (LMICs), including Mali, often face challenges such as limited access to comprehensive health information and services. Mobile health (mHealth) interventions, particularly SMS-based interventions, have shown promise in addressing maternal health challenges. This review aims to provide an overview of existing SMS-based antenatal care (ANC) applications and assess their effectiveness in improving maternal and child health outcomes.

**Design:**

A systematic literature review was conducted based on updated PRISMA 2020 guidelines.

**Data sources:**

PubMed, Scopus, Web of Science, Cochrane Library, Association for Information Systems eLibrary, Direct Science and Google Scholar were searched through 25 March 2024.

**Eligibility criteria:**

Studies that focused on SMS-based interventions designed to improve ANC information and attendance, published in English or French, conducted in LMICs and published between 2014 and 2024 were included. Exclusion criteria eliminated studies that did not report primary outcomes or did not directly involve SMS-based interventions for ANC.

**Data extraction and synthesis:**

Relevant data were systematically extracted, including study characteristics, intervention details, and outcome measures. The risk of bias was assessed using the Cochrane Risk of Bias tool for randomised trials (RoB 2), the Risk Of Bias In Non-randomised Studies-of Interventions (ROBINS-I) and the Checklist for Reporting the Development and Evaluation of Complex Interventions in Healthcare (CReDECI), depending on the study design. A subgroup analysis was performed to explore variations in outcomes by region and study design.

**Results:**

The review identified a range of SMS-based interventions (n=12) that differed in target audience, message frequency (weekly, pregnancy stage-oriented) and content (reminders (91.7% of cases, 11/12), educational (75%) and danger signs (16.7%)). Regional analysis highlighted significant research activity in East Africa but with mixed significance levels. The study design analysis revealed that randomised controlled trials yielded the most significant results, with five of eight studies showing full significance, whereas quasi-experimental studies demonstrated consistent but less frequent effectiveness. Implementation tools varied from SMS gateways to custom applications and third-party platforms, with some interventions combining these approaches. SMS interventions positively impacted ANC attendance, maternal health knowledge and behaviours, with effectiveness varying based on the intervention type, content, frequency and implementation approach.

**Conclusion:**

SMS-based interventions have the potential to enhance ANC in LMICs by providing tailored health information and promoting healthy behaviours. Further research should focus on refining or replicating these interventions and exploring their long-term effects on maternal and child health outcomes, particularly in underrepresented regions.

**STRENGTHS AND LIMITATIONS OF THIS STUDY**

- This review used the Preferred Reporting Items for Systematic Reviews and Meta-Analyses 2020 guidelines, ensuring a thorough and standardised approach to conducting the systematic review, thereby enhancing the transparency and reproducibility of the research process.
- The risk of bias in the included studies was meticulously assessed using three robust tools: RoB 2, ROBINS-I and the CReDECI.
- Data extraction and synthesis followed the predefined criteria to enhance the consistency and reliability.
- A notable limitation is that only one reviewer assessed the included papers.
- Quantitative statistical analysis typically performed in meta-analyses, such as pooled effect size calculation, was not undertaken, as the study was limited to a systematic review to inform our research focus.

## Introduction

 The lack of comprehensive health information and services for pregnant women is a significant challenge in improving maternal and child health in Mali and similar settings. Literature reports that knowledge of the place of consultation, treatment costs, pregnancy complications and the place of antenatal care (ANC) treatment influence maternal mortality.^1^ Additionally, regarding services, births attended by skilled health personnel correlate with maternal mortality in sub-Saharan Africa.^2^ Poor antenatal and maternal health awareness among pregnant women contributes to inadequate health behaviours and care-seeking, causing avoidable morbidity and mortality.

ANC is a critical component of maternal healthcare that aims to monitor and enhance the health outcomes of pregnant women and their unborn children. Regular ANC visits enable healthcare providers to detect and manage potential health problems, educate women about pregnancy and childbirth and advocate for healthy behaviours that benefit both the mother and the child.^3^,^5^ Despite the global recognition of ANC’s importance, significant challenges persist in ensuring comprehensive care for all pregnant women, particularly in low- and middle-income countries (LMICs). Studies have shown that maternal education, household income and cultural beliefs significantly affect the utilisation of ANC services, with disparities in access and use across different socioeconomic and demographic groups.^5 6^ Addressing these challenges requires targeted interventions to improve the access, awareness and affordability of ANC services for pregnant women in these regions.

The rapid growth of mobile technology has led to innovative ways of increasing healthcare access and engaging patients. SMS-based systems have become vital for closing information gaps and boosting engagement with ANC services. These applications offer a platform for delivering timely, relevant information directly to the mobile phones of pregnant women, thus increasing awareness of the importance of ANC, reminding women of their upcoming appointments and providing crucial health-related guidance.^7^,^12^ Studies have demonstrated the potential of mobile health (mHealth) interventions to monitor prenatal care among pregnant women in LMICs^13^ and have evaluated the effectiveness of SMS on focused ANC visits and skilled birth attendance in such settings.^7^

For instance, a meta-analysis found that mHealth interventions improved the uptake of four or more ANC visits among pregnant women in LMICs, with both one-way and two-way communication methods showing positive effects.^14^ SMS support during pregnancy was also associated with a decreased risk of perinatal death compared with routine prenatal care in one study.^15^ Interestingly, while SMS interventions generally improved ANC utilisation, their impact varied across contexts. In settings where facility delivery rates were already high, SMS interventions showed unclear effects. However, in areas with lower facility delivery rates, these interventions significantly increase facility-based deliveries.^14^

Despite rapid advancements in mobile health technologies, basic SMS remains a cornerstone in regions where limited internet access and low smartphone penetration hinder the adoption of complex systems. This review addresses the utility and effectiveness of SMS-based interventions in settings in which basic utilities such as electricity or the internet may be unreliable. By exploring the impact of SMS-based applications on metrics such as ANC visit attendance and skilled delivery attendance, we aim to clarify the potential of digital interventions to complement traditional ANC services and contribute to reducing maternal and neonatal morbidity and mortality, supporting public health goals^16^ and contributing to the broader global health narrative of health, sustainability and transformation.^17^

The remainder of this paper is organised as follows. The second section details the methodology by describing the research question, data sources, search strategy, selection criteria and data extraction process. It also presents the analysis tools, data characteristics and risk-of-bias assessment. The third section presents the results, which are discussed in section four along with limitations. Section five concludes the paper.

## Methodology

A systematic approach was employed to identify and evaluate significant findings concerning the use of SMS-based interventions to improve ANC in developing countries, as documented in peer-reviewed online French and English journals over the past decade. To ensure a thorough and effective review process, we followed the updated guidelines outlined in the 2020 edition of the Preferred Reporting Items for Systematic reviews and Meta-Analyses (PRISMA).^18^ The PRISMA 2020 Abstracts and Checklist items can be accessed from the online supplemental appendix. The review process, including screening, quality assessment and data extraction, was conducted by a single reviewer due to resource constraints and the need for language proficiency. To minimise potential bias, predefined inclusion and exclusion criteria were strictly followed, and standardised tools, such as RoB 2, ROBINS-I and ROBVIS, were applied to ensure methodological rigor.

### Research questions

The objectives of this study were to address the following research questions:

RQ1: What are the characteristics and availability of SMS-based applications developed between 2014 and 2024 to enhance ANC information and attendance among pregnant women in LMICs?

RQ2: How effective are these SMS-based applications in improving ANC information and attendance among pregnant women in LMICs compared with the usual care?

#### Data sources

The search included the following electronic databases or search engines: PubMed (last searched 19 March 2024), Scopus (last searched 21 March 2024), Web of Science (last searched 22 March 2024), Cochrane Library (last searched 20 March 2024), Association for Information Systems eLibrary (AISeL) (last searched 20 March 2024), Direct Science (last searched 21 March 2024) and Google Scholar (last searched 25 March 2024). These searches were conducted to ensure the inclusion of the most up-to-date and relevant literature.

#### Search strategy

**Figure 1:**
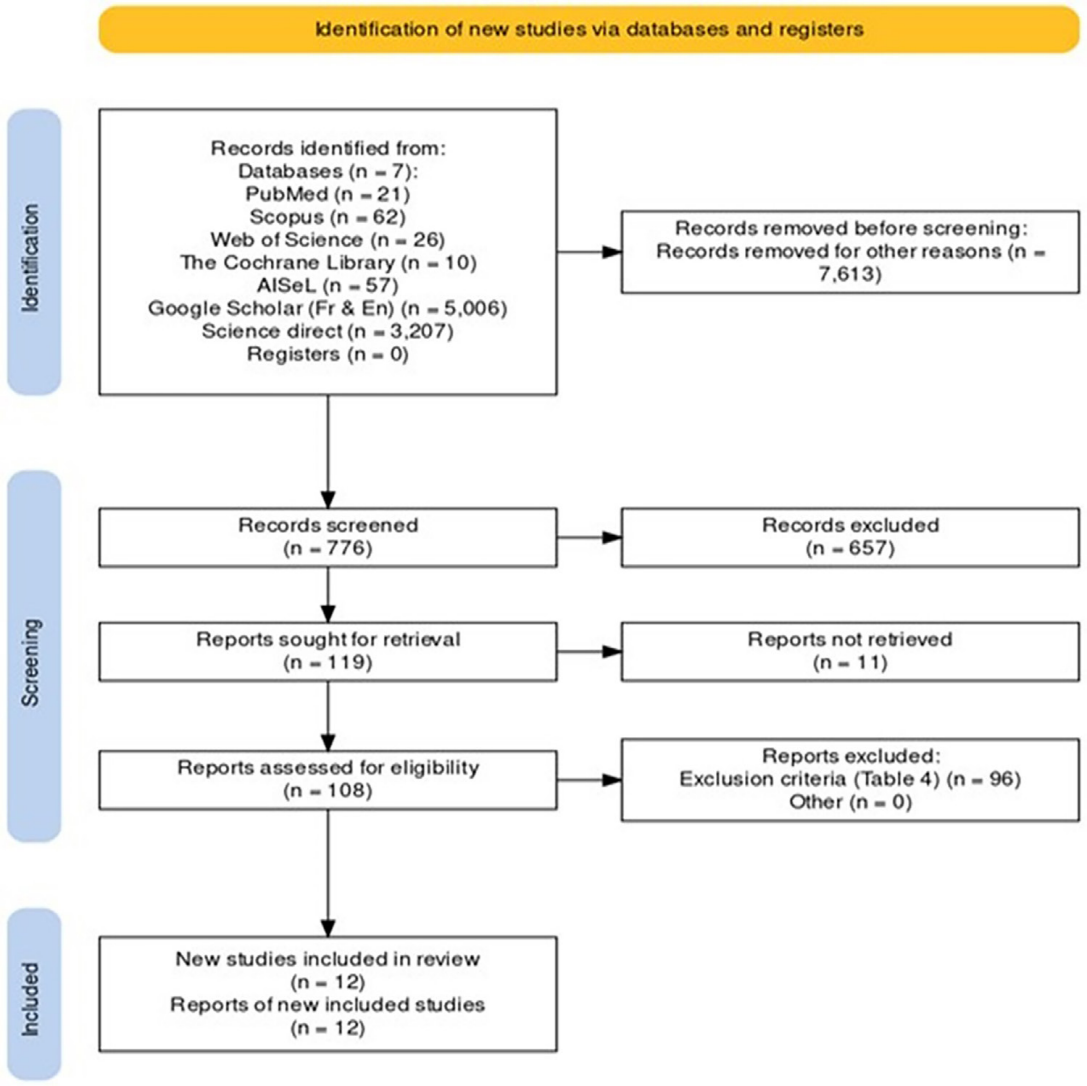
Flow diagram of the search.

The formulated research questions guided the construction of the search strings, leading to their combination through logical connectors. The resulting string was [(“SMS-based applications” OR “text messaging” OR “mobile health” OR “mHealth”) AND (“antenatal care” OR “prenatal care” OR “pregnancy care” OR “ANC”) AND (“developing countries” OR “low-income countries” OR “resource-limited settings”)]. This process was adapted according to the requirements of each electronic database. Science Direct, for example, did not accept more than eight logical connectors in a single search. The author translated the search string into French by combining words and expressions used in the English search. The resulting string was (*“applications basées sur SMS” OU “messagerie texte” OU “santé mobile” OU “mSanté”) ET (“soins prénatals” OU “soins anténataux” OU “soins pendant la grossesse” OU “CPN”) ET (“pays en développement” OU “pays à faible revenu” OU “contextes à ressources limitées”*). The process used for searching and selecting different publications is summarised in a Diagram Flow and presented in figure 1. The flow diagram of the search was created using the R-developed online tool by Haddaway *et al*.^19^ Online supplemental eTable 1 summarises the full search strategy, and online supplemental eTable 2 details the results per database.

#### Selection criteria

Initially, 776 publications were found, as detailed in online supplemental eTable 2. Additional inclusion and exclusion criteria were applied to shift the initial findings to pinpoint studies pertinent to our goals. Consequently, these publications underwent a rigorous screening process based on the inclusion and exclusion criteria. These criteria were defined to ensure the relevance and quality of the analysed data. The study design criteria included randomised controlled trials (RCTs), quasi-experimental, observational and qualitative studies that provided data on the implementation, usage and outcomes of SMS-based ANC interventions. Editorials, reviews, opinion pieces and studies lacking primary data or clear outcomes related to ANC and SMS-based interventions were excluded.

The population criteria focused on studies involving pregnant women in LMICs encompassing women of all ages, ethnicities and stages of pregnancy. For the intervention criteria, the studies needed to focus on SMS-based systems designed to improve ANC information and attendance. These included interventions promoting health education, appointment reminders, health monitoring and support through text messaging. Studies that did not specifically use SMS-based communication as the primary method for delivering ANC information or support were excluded. Criteria such as comparators, outcomes, publication dates and languages were also used. Online supplemental eTable 3 provides a detailed description of the inclusion and exclusion criteria, along with the rationale for each criterion. In the subsequent phase, the process involved verifying the presence of duplicate papers given that multiple databases were used for the search. This resulted in identifying and removing 11 duplicate documents from the dataset. Full texts of papers were then retrieved and checked. Following this meticulous selection phase, a final count of 12 papers was deemed appropriate and suitable for review (figure 1).

#### Data extraction

After completing the selection process, we extracted information from the selected papers. The study identification items included author names, paper title, journal, publication year, study design type and the country where the study was conducted. Details regarding the study participants were also extracted, including an accurate description of the study population, sample size and primary inclusion and exclusion criteria. Information on the intervention details extracted includes a description and purpose of the SMS-based application as presented in the paper, the content of messages, frequency of sending, resources and tools for implementation and intervention duration. Additionally, control or comparator interventions were retrieved as reported, if applicable. The reported outcomes (primary and secondary) were then extracted. Key findings related to the indicated outcomes, statistical significance where applicable and any reported limitations were also extracted. The complete data extraction form is provided in online supplemental eTable 4.

#### Tools and analysis

The data set was managed using the open-source desktop-based application Mendeley version 1.19.8. The extracted items were stored and used to generate descriptive statistics using JabRef (version 5.13), Microsoft 365 Excel (version 2403) and IBM SPSS Statistics 20.

#### Patient and public involvement

None.

#### Data characteristics

A bibliometric overview of the selected papers is described in table 1. Each paper was assigned a numerical identifier and categorised according to the year of publication, from oldest to most recent and by source.

**Table 1:** Bibliometric overview

ID	Author(s)	Title	Journal/Conf	Country (region)	Year	Source
01	Lund *et al*^12^	Mobile Phone Intervention Reduces Perinatal Mortality in Zanzibar: Secondary Outcomes of a Cluster Randomized Controlled Trial	JMIR mHealth and uHealth	Tanzania (Zanzibar)	2014	PubMed
02	Masoi & Kibusi^9^	Improving pregnant women’s knowledge on danger signs and birth preparedness practices using an interactive mobile messaging alert system in Dodoma region, Tanzania: a controlled quasi-experimental study	Reproductive Health	Tanzania (Dodoma)	2019	
03	Nuhu *et al*^8^	Impact of mobile health on maternal and child health service utilization and continuum of care in Northern Ghana	Scientific Reports	Ghana	2023	
04	Alhaidari *et al*^27^	Feasibility and acceptability of text messaging to support antenatal healthcare in Iraqi pregnant women: A pilot study.	Journal of Perinatal Medicine	Iraq	2018	Scopus
05	Ronen *et al*^10^	Evaluation of a two-way SMS messaging strategy to reduce neonatal mortality: rationale, design and methods of the Mobile WACh NEO randomised controlled trial in Kenya.	BMJ Open	Kenya	2021	AISeL
06	Batool *et al*^33^	Maternal complications: Nuances in mobile interventions for maternal health in urban Pakistan	Proceedings of the Ninth International Conference on Information and Communication Technologies and Development	Pakistan	2017	Google Scholar
07	Atnafu *et al*^28^	The role of mHealth intervention on maternal and child health service delivery: findings from a randomized controlled field trial in rural Ethiopia	mHealth	Ethiopia	2017	
08	Omole *et al*^31^	The effect of mobile phone short message service on maternal health in south‐west Nigeria	The International Journal of Health Planning and Management	Nigeria	2018	
09	Thompson *et al*^32^	Connecting mothers to care: Effectiveness and scale-up of an mHealth program in Timor-Leste.	Journal of Global Health	Timor-Leste	2019	
10	Muhoza *et al*^24^	A mobile-based technology to improve male involvement in antenatal care.	Kabale University Interdisciplinary Research Journal	Uganda	2022	
11	Oliveira-Ciabati *et al*^34^	SISPRENACEL - MHealth tool to empower PRENACEL strategy.	Procedia Computer Science	Brazil	2017	Science Direct
12	Kawakatsu *et al*^35^	Cost-effectiveness of SMS appointment reminders in increasing vaccination uptake in Lagos, Nigeria: A multi-centered randomised controlled trial	Vaccine	Nigeria	2020	

#### Risk-of-bias assessment

In this study, the dataset comprised 12 scholarly articles. Each article was evaluated for potential bias, with assessment criteria varying according to the study design. Three distinct tools were used to conduct this assessment: version 2 of the Cochrane Risk-of-Bias tool for randomised trials (RoB 2)^20^ was applied to eight studies, the Risk Of Bias In Non-randomised Studies—of Interventions (ROBINS-I) tool^21^ to three studies and the Checklist for Reporting the Development and Evaluation of Complex Interventions in Healthcare^22^ were conveniently used for one study. Visual representations of the assessments, including traffic light plots (see onlinesupplemental eFigure 1^2^) and summary plots (see online onlinesupplemental eFigures 3^4^), were created for the two groups (RoB two and ROBINS-I). Refer to online supplemental eFigures 5 to the assess the study using the checklist. These plots were generated using the Risk Of Bias VISualisation tool.^23^ The overall risk assessment for the papers was categorised as ‘some concerns’. Consequently, we did not exclude any of the documents included due to the absence of many significant high/critical issues with individual papers.

## Results

### SMS app inventory (RQ1)

#### Overview of apps

The dataset consists of 12 applications. These ranged from basic, one-way SMS-sending apps to more complex, bidirectional communication platforms that connect pregnant women with healthcare providers throughout and sometimes beyond the pregnancy period. Table 2 provides an overview of the identified apps and offers details on each app’s target population, key features and study design employed to evaluate its effectiveness. App names are given where the authors gave specific names to their developed apps.

**Table 2:** Overview of apps

App name	Country (region)	Target population	Key features	Study design
The Wired Mothers	Tanzania (Zanzibar)	Pregnant women attending ANC at 24 primary healthcare facilities across six districts on the island of Unguja	Unidirectional text messaging A mobile phone voucher system for two-way communication between pregnant women and their primary healthcare providers.	Pragmatic, cluster RCT
N/A	Tanzania (Dodoma)	Pregnant women	Provide simple health education (obstetric danger signs, newborn danger signs, individual birth preparedness, complication readiness) Engage expecting parents (mother and father) with essential health information. Two-way communication	A quasi-experimental study with a control group is characterised explicitly as a ‘pre- and post-test with a control group’.
T4MCH	Ghana	Pregnant women	Automated messaging (SMS/voice messages)	Standard guidelines for reporting quasi-experimental studies using the Transparent Reporting of Evaluations with Non-randomised Design/Quasi-Experimental Study Design (TREND)
N/A	Iraq	Pregnant women attending an antenatal clinic linked to Al Elwiya Maternity Teaching Hospital	Automated SMS	Controlled experimental study
Mobile WACh NEO system	Kenya	Pregnant women were recruited from four different facilities in Kenya.	Two-way communication Automated messaging Support for multiple languages Response management Participant tracking Cost-free for participants	RCT
N/A	Pakistan	Pregnant women enrolled in the trial conducted at Female Willingdon Hospital in Lahore	Multi-modal communication (SMS and automated voices) Automated delivery Data tracking	RCT
Customised FrontLineSMS	Ethiopia	Women aged 15–49 years who had at least one child	Automated messaging Data exchange between CHW and CHW Contraceptive stock management	Community-based RCT
Maternal Health Plus	Nigeria	Pregnant women attending ANC within the Ife-Ijesa zone.	Automatic delivery of SMS Two-way communication Database management Language preference	RCT
Liga Inan	Timor-Leste	Women aged 15–49 years with a child up to 24 months of age.	Web-based platform connected to a GSM Automatic delivery of SMS Voice communication	Quasi-experimental design.
N/A	Uganda	Pregnant women and their partners	Cloud-based platform Monitoring ANC-seeking behaviour Automatic delivery of SMS	Pragmatic randomised trial
SISPRENACEL	Brazil	Pregnant women	Automatic delivery of SMS Two-way communication Individualised interaction management (chat-like format) Researcher access Private cloud deployment	A socio-technical approach using the prototype method.
N/A	Nigeria	Pregnant women	Automatic delivery of SMS Customisation (depending on the type of health service) Cloud server Unique QR code for each user	Multi-centred RCT

ANCantenatal careCHWcommunity health workerGSMGlobal System for Mobile CommunicationsN/A, Not AvailableRCTrandomised controlled trial

#### Detailed app descriptions

This subsection comprehensively describes each application based on extracted data. Essential intervention details, such as message content, sending frequencies and the development tools used (see table 3), are provided.

**Table 3:** Detailed app descriptions

Study ID	Content of messages	Frequency	Tools/resources employed for implementation	Duration of the intervention
01	Health education on danger signs in pregnancy, the importance of skilled delivery attendance and reminders for upcoming antenatal care (ANC) visits.	The frequency of the messages varied throughout the pregnancy, with an increase in frequency to weekly messages during the last 4 weeks before delivery.	Specific software name or platforms used for development is not mentioned.	The study followed the women until 42 days post-delivery to assess the impact of the mobile phone intervention on perinatal outcomes.
02	Obstetric and newborn danger signs and birth preparedness and complication readiness.	First trimester: one message per week.Second trimester: two messages per week.Third trimester: three messages per week.	Specific software name or platform used for development is not mentioned	From the initial ANC visit until the point of delivery
03	The messages include the importance of regular ANC visits, the benefits of facility-based deliveries, and the necessity of postnatal care.	Weekly	Savana signatures: design and execution of the project;Salasan Inc: technological framework;Mustimuhw Information Solutions: software solutions	1 August 2017 to 30 September 2017.
04	General health messages,Reminders to visit PHCC,Nutritional advice,Lifestyle education.	Weekly, every Friday between 4 PM and 6 PM	forat-sms.com: bulk messaging platform	Not specified
05	Critical information on pregnancy, birth planning, infant care, and emergency responses	From delivery to 2 weeks postpartum, mothers get two daily messages to bolster care practices and offer continuous support.	Detailed in another paper^37^	From enrolment at 28–36 weeks of gestation until 6 weeks postpartum
06	Information about prenatal care, reminders for ultrasound tests, encouragement to follow medical advice and attend scheduled appointments.	It is not specified, but it is mentioned that the app could manage diverse messaging needs across distinct stages of pregnancy.	SMS Service Provider: API SMSAll.pkTelephony software: for automated calls, Asterisk was used, coupled with a Primary Rate Interface (PRI) line to manage multiple concurrent calls.	2 months
07	ANC reminders andChild immunisation	Health extension workers (HEWs) received ANC appointment reminders at gestational weeks 14, 24, 30 and 36. Vaccination appointment reminders were sent at 6, 10 and 14 weeks, and 9 months. HEWs then sent a reminder 1 week prior to monthly vaccinations.	Mobile phones equipped with customised FrontLineSMS and Central server and Local network and Short-code System and GSM Modem subscription	September 2012 to October 2013: 13 months
08	Clinic reminders, specific pregnancy-related health tips, general tips	Delivered periodically, based on the ANC appointment schedule of each participant.	Mobile devices, SMS Enabler version 2.5.5, A MySQL database	December 2013 to December 2014
09	Reminders for care-seeking and promoted safe pregnancy and delivery practices.	Messages were sent twice weekly, precisely every Monday and Thursday.	Mobile devices, web-based applications connected to a GSM gateway	2 years
10	Appointment reminders	Weekly	A cloud-based platform, AfricasTalking API.	9 months
11	Information on ANC, pregnancy, and delivery topics.	Not specified but likely according to pregnancy stages.	Client-server architecture, CakePHP and MySQL for data storage, AdminLTE version 1.0 for GUI.	April 2015 to May 2016
12	Visit reminder messages.	SMS text reminder 2 days before their scheduled appointments. If clients did not attend their appointments, an additional reminder was sent 7 days after the original appointment date as a defaulter tracing measure.	Mobile application linked to a cloud server, with a unique QR code for each user.	first April to 30th June 2019

GUI, Graphical User Interface; code, quick-response code; RCT, randomised controlled trial

### Effectiveness Evidence (RQ2)

#### Overview of studies

Among the 12 studies, six primary outcomes were identified and further classified into effectiveness and safety domains, as well as primary and secondary categories. **Primary** effectiveness outcomes included improved attendance (n=9) and skilled delivery attendance (n=4). The primary safety outcomes included a reduction in neonatal mortality (n=1) and reduced complications (n=1). Secondary effectiveness outcomes included increased knowledge (n=3) and patient satisfaction (n=1). Online supplemental eTable 5 provides a detailed breakdown of these outcomes categorised by study design.

Regarding the message content sent to participants (including women and, in one case, their male partners^24^), the key themes revolved around appointment reminders (observed in 11 studies, representing 91.7% of cases), educational content (75% of cases), emergency or danger alerts (16.7% of cases) and combinations of these themes (66.7% of cases).Online supplemental eFigure 6 illustrates the frequency of SMS content types across the different apps. For the detailed content types per study, please refer to table 3.

The SMS-sending frequency was consistent across the studies. In five studies, messages were sent weekly, while in other cases, the frequency was adjusted according to the pregnancy stage or specific contextual timing. For instance, in the setup described by Masoi and Kibusi,^9^ the frequency varied by pregnancy stage: one message per week during the first trimester, two per week in the second trimester and three per week in the third trimester. These variations were noted in eight studies, including some that used weekly SMS during certain phases or daily SMS from delivery to 2 weeks postpartum.^10^ A common trend in apps using varied frequencies was a systematic increase in message intervals as the delivery date approached. Table 2 provides detailed information on each application. The intervention durations varied, with some lasting less than 3 months (16.7%), others ranging from 3 to 6 months (16.7%), some spanning 6 to 12 months (41.7%) and three studies exceeding 12 months (25%).

The breakdown of development tools or approaches for app implementation is as follows. An SMS gateway (26.67% usage rate among evaluated apps) facilitates the efficient delivery and receipt of text messages, making it suitable for large-scale messaging campaigns due to its simplicity.^25^ Custom apps (36.67%) provide personalised features like interactive messaging and data analytics, demanding substantial development resources while offering significant customisation. Third-party platforms (16.67%) are pre-built solutions with scheduling and often analytics features but may lack the flexibility of custom apps. A combined approach (20% usage rate) combines the strengths of multiple tools, such as using a custom app for analytics along with an SMS gateway or a third-party platform for messaging, allowing for simplicity, customisation and scalability tailored to various SMS interventions.^26^ The specific names and/or platforms used by each app (when provided in the article corpus) are listed in table 2.

#### Study findings

Unsurprisingly, all studies highlighted the significant impact of SMS-based interventions on maternal healthcare. Lund *et al*^12^ discovered a substantial rise in ANC attendance, with women adhering to the WHO recommendations for four or more visits. The same has been observed in other studies.^8 24 27 28^ Moreover, they^12^ observed an increase in skilled delivery attendance among urban women, with an OR of 5.73 (95% CI: 1.51 to 21.81). Notably, it significantly reduced perinatal mortality, with an OR of 0.50 (95% CI: 0.27 to 0.93). Ronen *et al*,^10^ in the pilot phase^29^ of their ongoing randomised controlled study (Mobile WACh NEO RCT), identified that among women residing in areas with elevated rates of stillbirth, perinatal and infant mortality, increasing maternal age was the sole predictor of stillbirth. It is essential to highlight that although we included their main study in our dataset, the results have not yet been compiled and published as of the writing of this paper. The trial concluded with participant enrolment (5020 participants) on 30 June 2022, and follow-up was scheduled to continue until February 2023.^30^ Consequently, we relied on the pilot-phase results.^29^ Table 4 shows the different studies along with the effect sizes and statistical significance of their primary outcomes, as reported in the content of the papers.

**Table 4:** Effectiveness evidence

Study	Main outcomes and significance	Conclusion
^12^	Significant effect on antenatal care (ANC) attendance, with an OR of 2.39 and a 95% CI of 1.03 to 5.55.Increased skilled delivery attendance among urban women, with an OR of 5.73 and a 95% CI of 1.51 to 21.81.Significant reduction in perinatal mortality with the mobile phone intervention, with an OR of 0.50 and a 95% CI of 0.27 to 0.93.	The study illustrates that the mobile phone intervention effectively improved critical maternal health outcomes and significantly reduced perinatal mortality.
^9^	Significant increase in knowledge about obstetric and newborn danger signs (large effect size 85%).Higher scores in birth preparedness and complication readiness (effect size of 90%).	The significant effect sizes in both primary outcomes suggest that the intervention had a robust impact on the participants.
^8^	Increased ANC attendance, with an average treatment effect (ATE) of about 18%.Increase in the number of women opting for facility-based delivery (14%).PNC attendance also increased with the intervention (27%).	The results underscore the intervention’s positive effect on maternal health, notably increasing attendance and utilisation of essential maternal and child health services.
^27^	Over 85% of the participants in the intervention group expressed satisfaction with the SMS-based support.Statistically significant increase in the median number of ANC visits compared.	The intervention significantly increased engagement in ANC, and positive feedback was received from participants regarding satisfaction.
^29^	The stillbirth rate observed was sixteen per 1000 pregnancies.There were 17 neonatal deaths during the study period, leading to a neonatal mortality rate of 22 per 1000 live births.The perinatal death rate (including stillbirths and neonatal deaths up to 6 days of age) was 36 per 1000 pregnancies.	This pilot phase identified that among women residing in areas with elevated rates of stillbirth, perinatal and infant mortality, increasing maternal age was the sole predictor of stillbirth.
^33^	Significant improvements in knowledge about pregnancy and childbirth.No significant difference in the number of follow-up visits among the groups.	The study revealed substantial knowledge gains about pregnancy among participants, but the effect of increasing follow-up visits remained ambiguous due to social norms and logistical challenges.
^28^	Significant increase in the proportion of mothers attending more than four ANC visits in the intervention.Ezha (treatment 1): increased from 45.32% to 59.84%;Abeshge (treatment 2): increased from 15.8% to 31.5% ;Sodo (control): decreased from 24.48% to 23.27% ;P value: p<0.001 for Ezha and Abeshge.There was a significant increase in deliveries attended by skilled health workers in the intervention areas Ezha (treatment 1): increased from 26.79% to 55.23%;Abeshge (treatment 2): Increased from 41.96% to 63.54% ;Sodo (control): Increased from 21.79% to 52.05%.p<0.001 in Ezha, indicating robust improvement.	These findings highlight the improvements in healthcare services delivered to mothers and children due to the mobile intervention, with the most significant impact seen inANC attendance and skilled deliveries. However, limitations in the intervention’s effectiveness were noted in contraceptive utilisation and immunisation coverage.
^31^	There was a significant increase in the proportion of facility-based deliveries among the intervention (29%) and control groups (13%).96.6% of participants in the intervention group expressed support for the SMS intervention as a platform for maternal health promotion.	The intervention significantly improved maternal health behaviour by increasing the rate of facility-based deliveries among pregnant women.
^32^	No significant increase in the number of women receiving four or more ANC visits (OR=1.0 (95% CI: 0.54 to 0.9)).Significant increase in the likelihood of women having a skilled birth attendant present during delivery (OR=1.9 (95% CI: 1.1 to 3.2)).Significant increase in the likelihood of women delivering in a health facility (OR=1.9 (95% CI: 1.1 to 3.6)).	The Liga Inan programme significantly improved skilled birth attendance, facility deliveries, postpartum care and newborn health checks, though it did not notably affect ANC visits.
^24^	Increase in male involvement in ANC with a 50% adherence rate among male partners, meaning 10 out of the 20 male partners attended four consecutive antenatal visits.Improved ANC-seeking behaviour among pregnant mothers.	The results suggest that SMS-based interventions can positively impact male participation in ANC and improve pregnant mothers’ attendance rates.
^34^	The system received a high overall score of 6.33 out of 7 in usability, with the highest scores in system usefulness (6.61) and the lowest in information quality (6.03).High engagement with 22 296 scheduled SMS delivered, received 1249 messages from participants and 1823 SMS inquiries answered.The system could be adapted for national-level deployment	These results underscore the app’s effectiveness in achieving high user satisfaction and engagement and the potential for broader application in maternal health interventions.
^35^	Significant increase in the return rate for child vaccinations in the intervention group (4.8% to 6.0% higher return rate).No significant differences were observed in the return rates for ANC and family planning services between the intervention and control groups (adjusted ORs close to 1)	The results indicate that SMS reminders can enhance adherence to vaccination schedules, though their effectiveness may differ across health services, likely influenced by recipients’ perceived urgency or importance of the service.^35^

#### Subgroup analysis

Subgroup analysis explored the distribution and outcomes of the interventions across regions, study designs and intervention types, providing a better understanding of the factors influencing their effectiveness.

##### Regional distribution and significance

Regional distribution analysis revealed notable differences in the number of studies, outcomes and study-level significance across global regions. Five studies were conducted in East Africa (Ethiopia, Kenya, Tanzania and Uganda). Of these studies, four reported outcomes that were statistically significant,^9 12 24 28^ and one did not indicate significance (pilot).^29^ This highlights the region’s robust research activity. Three studies in West Africa (Ghana and Nigeria) emphasised the effectiveness of interventions in this region.^8 31^ Asia (Pakistan and Timor-Leste), the Middle East (Iraq), and South America (Brazil) are underrepresented with only one study per country.

##### Impact of study designs

RCTs dominated the dataset, with eight studies spanning East and West Africa and Asia. Of these, five demonstrated all outcomes as significant, while two reported partial significance (see online supplemental eTable 6). This reflects the robustness of the RCT design in yielding significant findings, although with some variations. Quasi-experimental studies, the second most common design, include three studies from Ghana, Tanzania and Timor–Leste. Two of these achieved full significance, while one fell under the ‘not applicable’ category. A sociotechnical approach using a prototype method is less common, as represented by a single study. It reports fully significant outcomes, indicating potential but limited generalisability due to their low frequency.

##### Effectiveness of intervention types

The intervention-type analysis revealed critical trends in the study’s effectiveness and applicability. Mixed interventions (educational and reminders) are the most prevalent, with six studies across diverse regions including Africa, Asia and the Middle East. Among these, five reported full significance, while one indicated partial significance. Educational messages, implemented in Brazil, Kenya and Tanzania, are associated with three studies, of which two demonstrated significant outcomes and one was categorised as ‘not applicable’. Reminders applied in Ethiopia, Nigeria and Uganda show similar proportions, with two studies achieving full significance and one partial significance (refer to online supplemental eFigure 7).

## Discussion

The findings underscore the potential of SMS-based interventions to enhance ANC attendance, maternal health knowledge and service utilisation in LMICs. Across the studies reviewed, SMS interventions demonstrated varying degrees of effectiveness (see table 4), reflecting diversity in implementation approaches, population contexts and healthcare systems.

Studies^12 28^ highlighted substantial improvements in ANC attendance and skilled delivery rates, with ORs and effect sizes indicating robust effects. These findings suggest that SMS reminders and educational messages can effectively address common barriers to maternal healthcare, such as a lack of awareness or forgetfulness. However, the mixed outcomes observed in some studies, such as^32^ who reported a limited impact on ANC visits despite significant improvements in skilled delivery and facility-based births, indicate the need for context-specific tailoring of message content and delivery frequency.

The review highlights the strong influence of SMS-based interventions on maternal health knowledge and birth preparedness. For instance, Masoi and Kibusi^9^ reported large effect sizes in knowledge about obstetric and newborn danger signs, while Batool *et al*^33^ emphasised knowledge gains despite the limited impact on follow-up visits. Effective interventions appear to combine timely reminders with actionable health education, reinforcing preparedness and engagement. Participant satisfaction was consistently high across studies such as Alhaidari *et al*^27^ and Oliveira-Ciabati *et al*,^34^ where users expressed positive feedback about the usability and relevance of SMS interventions. High engagement levels, including two-way communication and interactive features, were associated with better adherence to health recommendations. These results suggest that user-centred design and feedback mechanisms are critical to the success and sustainability of SMS interventions. However, interactive features in some cases might not be ideal in low-resource settings as it implies the use of advanced technologies (smartphones) that are not necessarily accessible to the targeted women.

Our subgroup analysis revealed regional, methodological and intervention-type variations in the effectiveness of the SMS-based ANC interventions. East Africa had the highest research activity, with most studies reporting statistically significant outcomes, whereas other regions, including West Africa, Asia, the Middle East and South America, were underrepresented. RCTs demonstrated the strongest evidence. Mixed interventions combining educational messages and reminders were the most effective, highlighting the importance of multifaceted approaches over stand-alone reminders or educational messages. These findings emphasise the need for further research in underrepresented regions and deeper exploration of intervention strategies to optimise SMS-based maternal health programmes.

Despite these positive findings, this review also revealed limitations in the effectiveness of SMS interventions. For instance, Kawakatsu *et al*^35^ reported variability in effectiveness across different health services, such as higher adherence to vaccination schedules but no significant improvement in ANC or family planning return rates. Others^33^ have identified logistical barriers and social norms as factors that limit follow-up visits. These mixed outcomes emphasise the need for comprehensive programme designs that account for broader systemic and sociocultural factors influencing maternal health behaviours.

Moreover, based on our risk assessment, most studies were categorised as having ‘some concerns’, with no studies excluded because of critical methodological flaws. While this suggests a moderate level of reliability, certain biases may still affect the interpretation of the results. For example, it^12^ exhibited high bias in two domains (D2: bias due to deviations from intended intervention and D4: bias in the measurement of outcomes), which may impact the validity of its reported reduction in perinatal mortality and maternal health improvements. Similarly, Muhoza *et al*^24^ had a high D2, suggesting potential concerns regarding deviations from the intended intervention (see online supplemental eFigure 1). In the case of^8 9^ serious bias due to confounding factors (D1, ROBINS-I) may influence the observed significant effect sizes in primary outcomes and maternal health benefits. Additionally, Thompson *et al*,^32^ who demonstrated improvements in skilled birth attendance and facility deliveries, had a serious concern with D5 (bias due to missing data), potentially affecting the reliability of their findings see online supplemental eFigure 2. The study,^34^ assessed with a checklist for reporting the development and evaluation of complex interventions in healthcare, was concerned with sustainability (D8), which may limit its long-term applicability (see online supplemental eFigure 5).

Despite these biases, the collective evidence supports the positive impact of SMS-based interventions on ANC attendance, maternal health outcomes and service utilisation. However, these findings should be interpreted with caution because of potential methodological limitations.

### Limitations and future research

Our study acknowledges several limitations that may influence the generalisability and applicability of the findings. This systematic review was not pre-registered in a database, which may be considered a limitation. However, as no clinical data were involved, registration was not mandatory. We ensured methodological transparency by outlining our search strategy, inclusion criteria and quality assessment approach. The review process was conducted by a single reviewer, which, despite ensuring a consistent approach, could introduce bias and limit the breadth of interpretation typically enriched by multi-reviewer analyses. Resource constraints and the availability of language-proficient subject matter experts necessitate this approach. To mitigate potential bias, rigorous adherence to predefined inclusion and exclusion criteria was maintained throughout the process. Although not optimal, this approach ensured the feasibility of the study within the available resources. Moreover, given that this study is focused solely on a systematic review, as stated, we did not conduct quantitative statistical analyses typically required for meta-analysis, such as pooled effect size calculations or heterogeneity tests (eg, prediction Intervals, or I²).^36^ While these methods could have added quantitative depth, they were not necessary to achieve the primary objective of synthesising and qualitatively analysing the evidence to inform our research focus. This methodological void should be addressed in future studies. Although we identified a concentration of studies from East Africa (5 of 12), this likely reflects the higher volume of SMS-based ANC interventions conducted and published in this region. Despite our comprehensive search strategy, studies from other LMICs may have been underrepresented or uncaptured, highlighting the need for further research in diverse geographical contexts to improve generalisability.

## Conclusion

This review shows that mobile health interventions hold significant promise for improving maternal health outcomes, particularly in LMICs (see online supplemental eFigure 8). The interventions demonstrated positive effects on ANC attendance, health knowledge and general maternal health behaviours, underscoring the value of digital health tools in resource-limited settings. However, the effectiveness of these interventions varied widely and was influenced by factors such as the content and frequency of messages and the implementation tools used. Continued efforts in this field can significantly reduce barriers to ANC and improve maternal and child health outcomes.

## supplementary material

10.1136/bmjopen-2024-089671 online supplemental file 1

10.1136/bmjopen-2024-089671 online supplemental file 2

10.1136/bmjopen-2024-089671 online supplemental file 3

10.1136/bmjopen-2024-089671 online supplemental file 4

10.1136/bmjopen-2024-089671 online supplemental file 5

10.1136/bmjopen-2024-089671 online supplemental file 6

10.1136/bmjopen-2024-089671 online supplemental file 7

10.1136/bmjopen-2024-089671 online supplemental file 8

10.1136/bmjopen-2024-089671 online supplemental file 9

10.1136/bmjopen-2024-089671 online supplemental file 10

## Data Availability

All data relevant to the study are included in the article or uploaded as supplementary information. Extracted data, both raw and coded, are available upon reasonable request from the corresponding author.
