# Supplementary figures and images for: Effectiveness of SMS-based interventions in enhancing antenatal care in developing countries: a systematic review
Source: BMJ Open. 2025 Feb 25;15(2):e089671. doi: 10.1136/bmjopen-2024-089671 (PMC11865757; doi:10.1136/bmjopen-2024-089671)

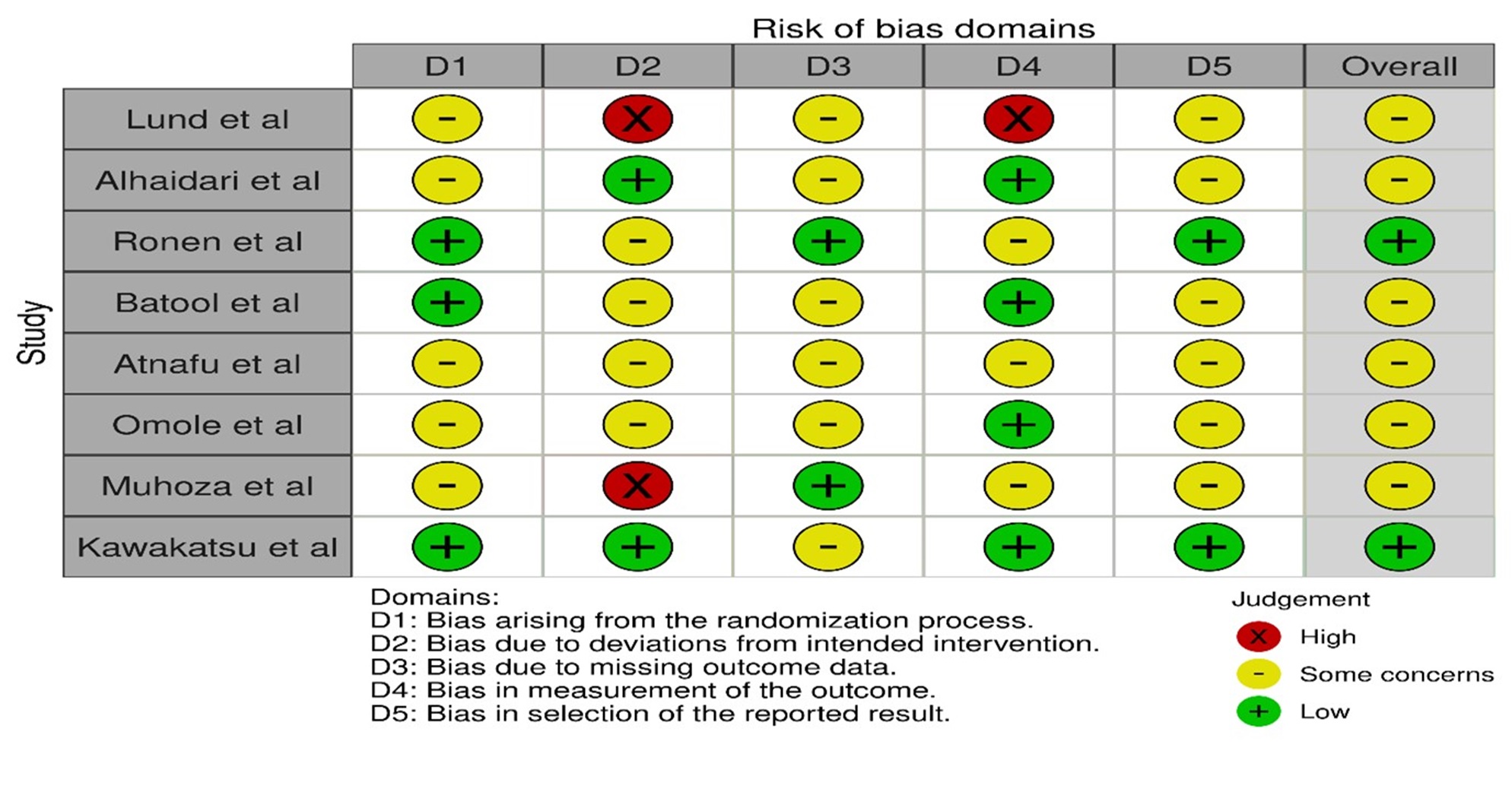

Supplement: online supplemental file 1 [file bmjopen-15-2-s001.jpg]

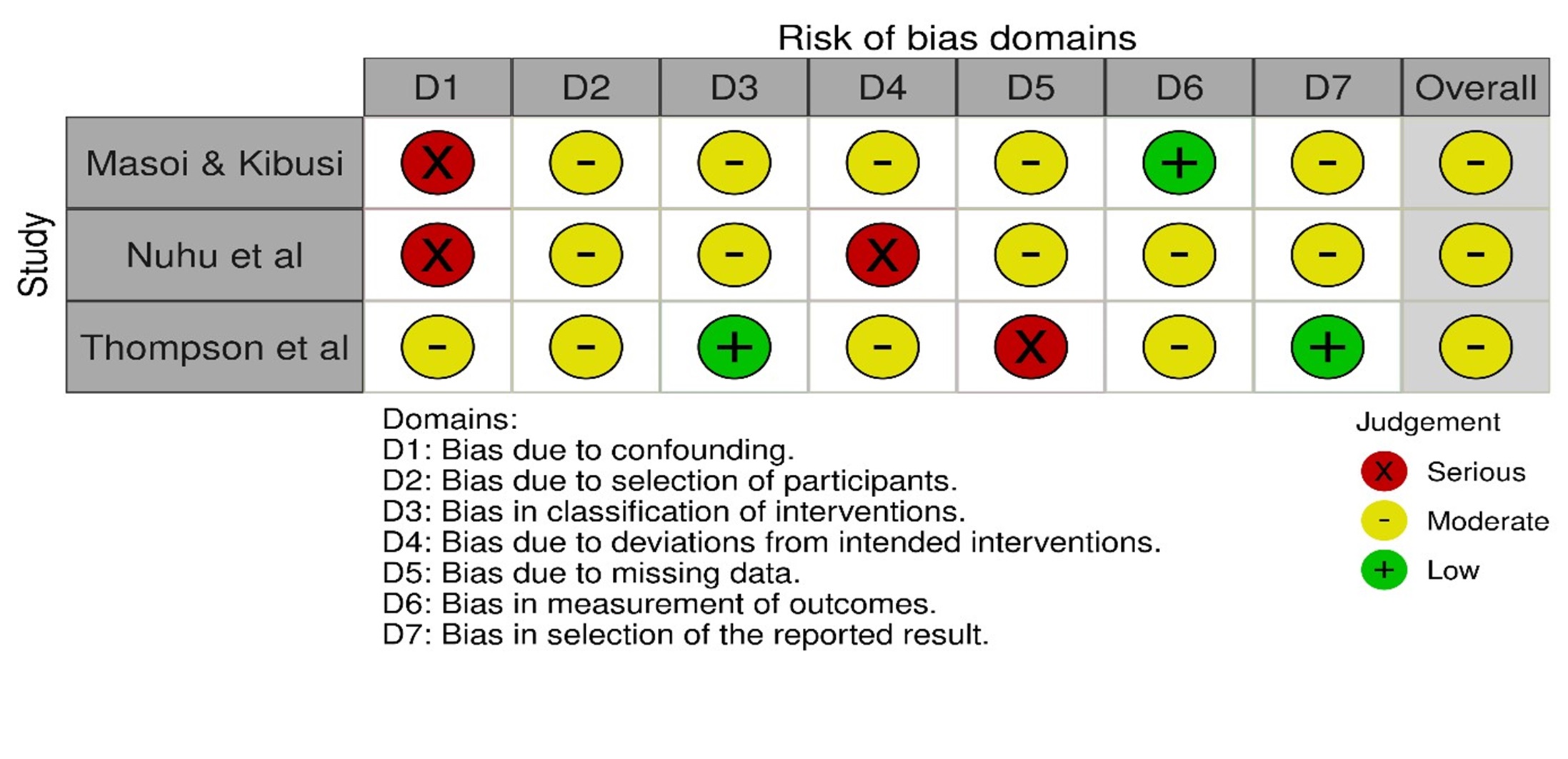

Supplement: online supplemental file 2 [file bmjopen-15-2-s002.jpg]

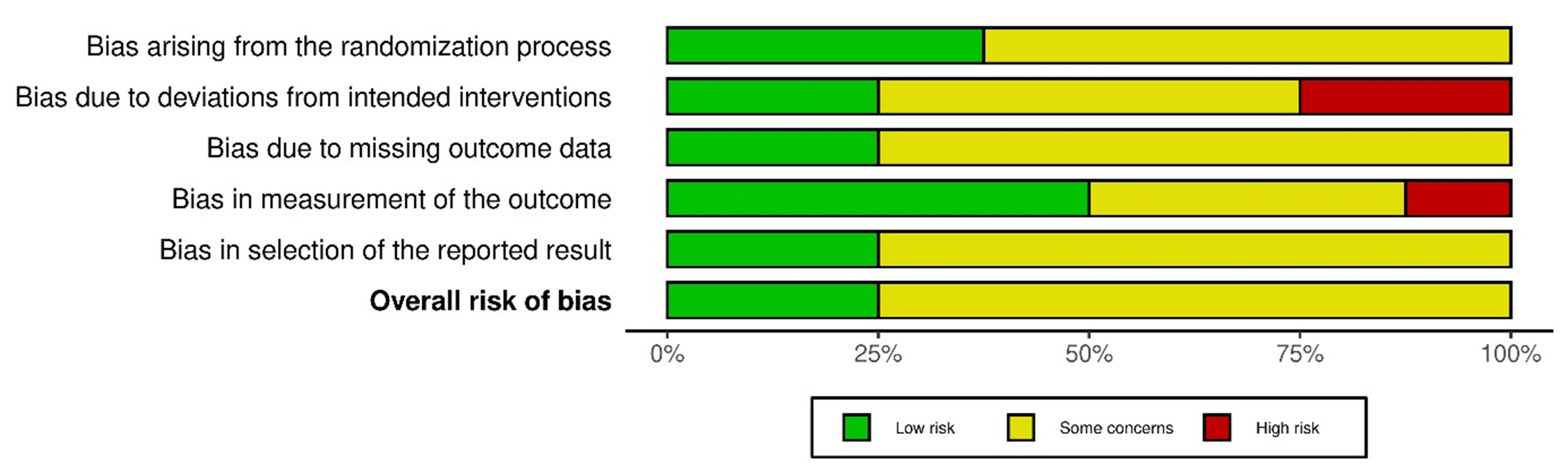

Supplement: online supplemental file 3 [file bmjopen-15-2-s003.jpg]

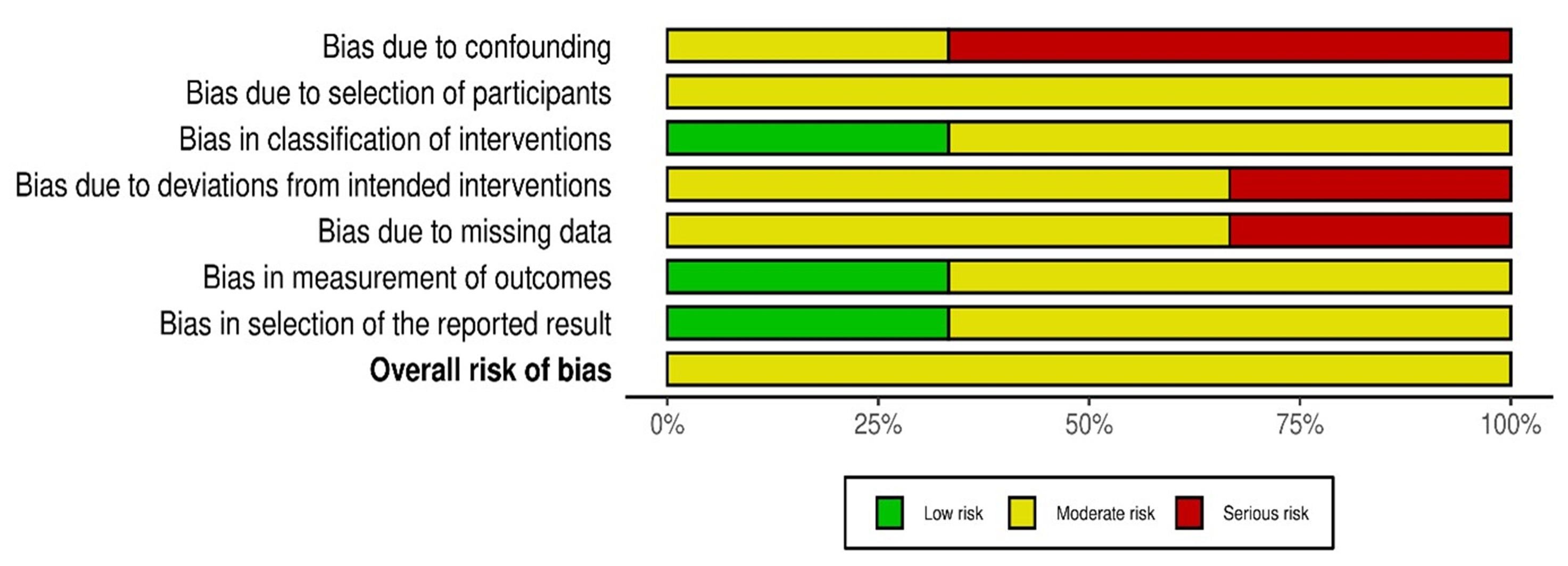

Supplement: online supplemental file 4 [file bmjopen-15-2-s004.jpg]

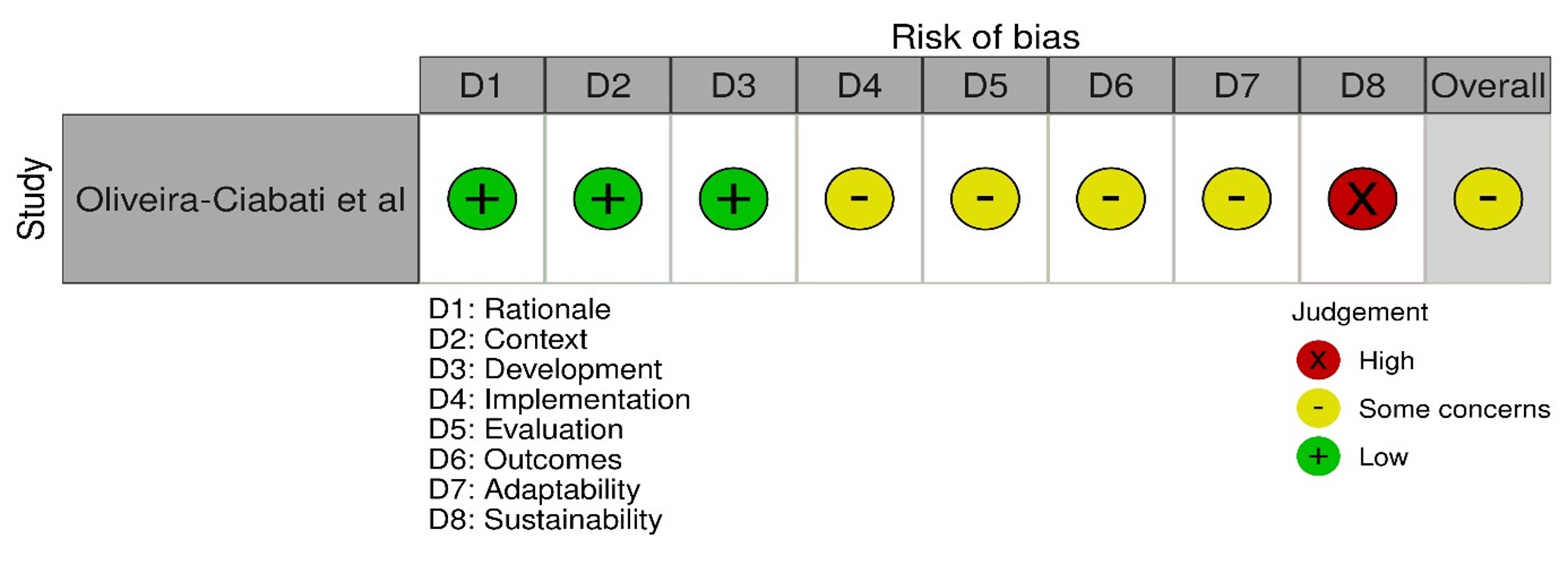

Supplement: online supplemental file 5 [file bmjopen-15-2-s005.jpg]

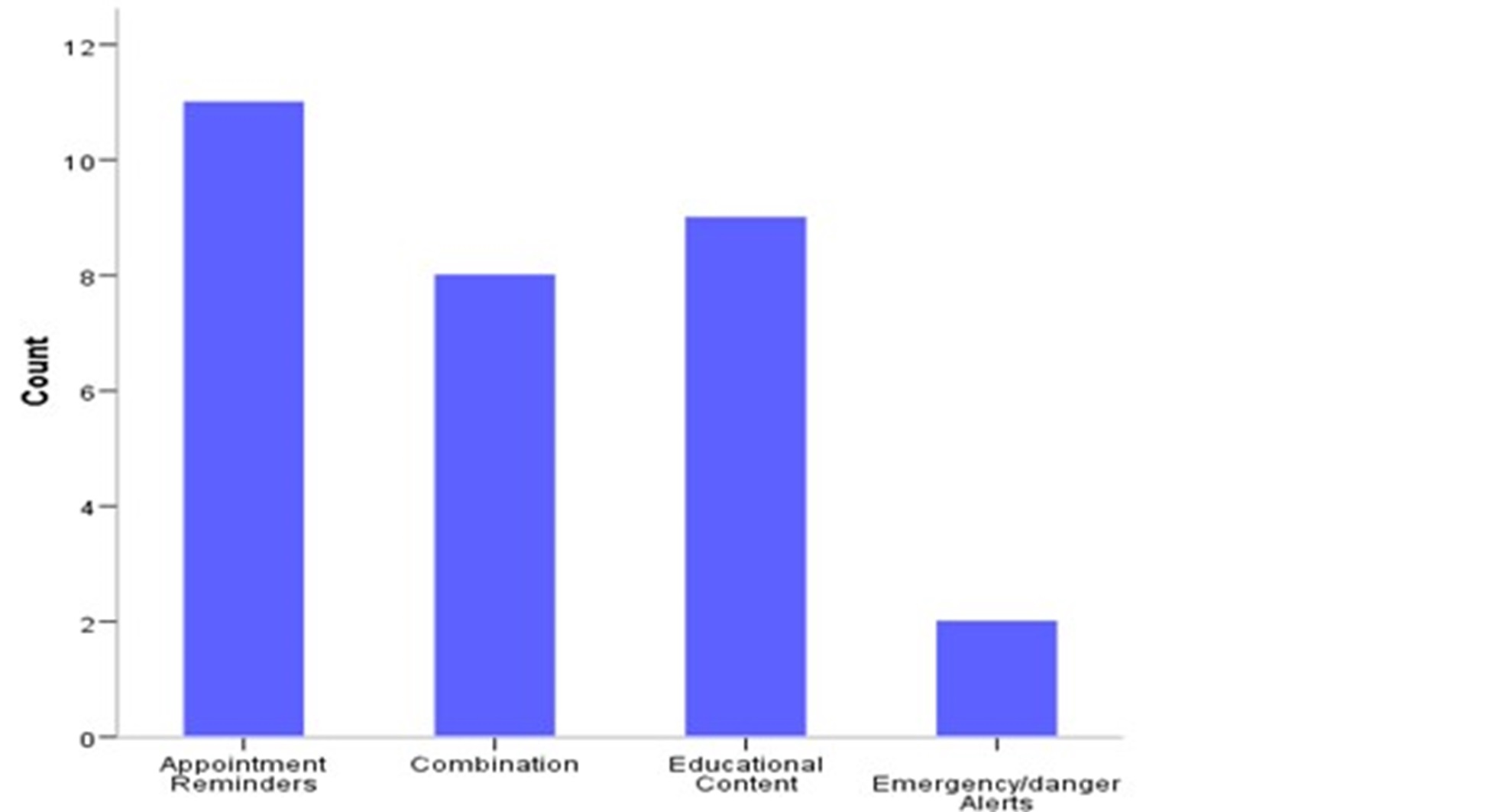

Supplement: online supplemental file 6 [file bmjopen-15-2-s006.jpg]

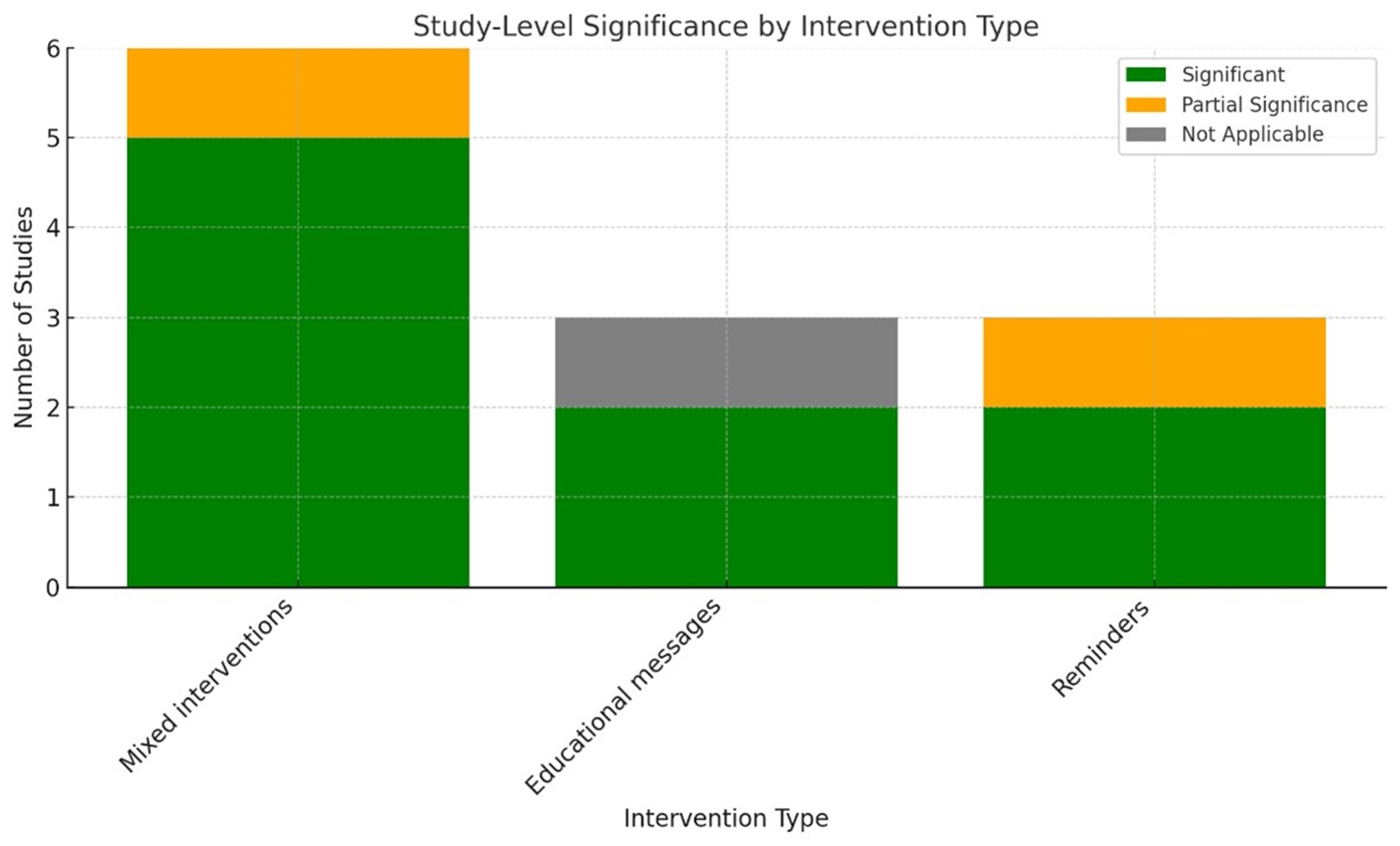

Supplement: online supplemental file 7 [file bmjopen-15-2-s007.jpg]

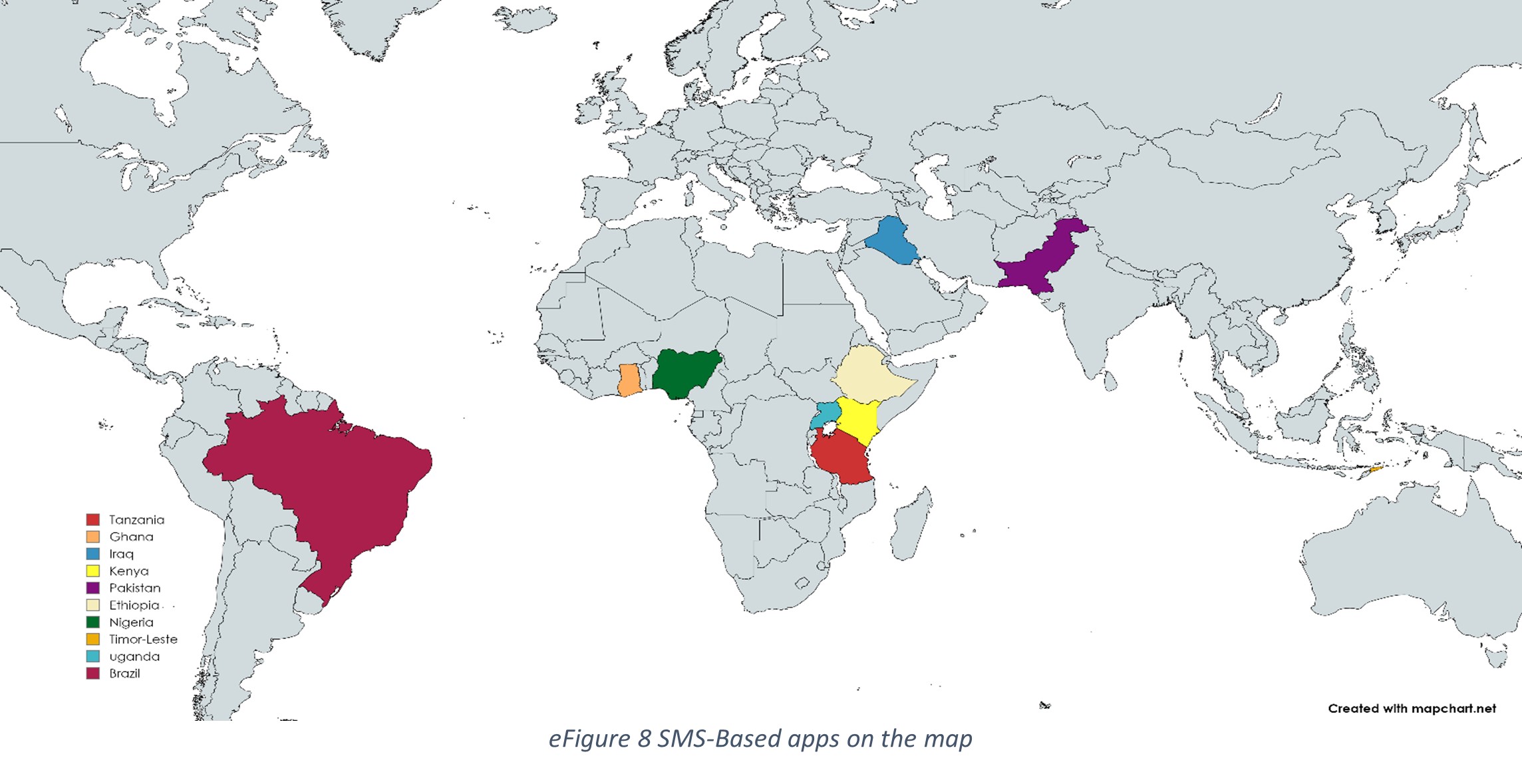

Supplement: online supplemental file 8 [file bmjopen-15-2-s008.jpg]
