# Supplementary material for: Effectiveness of SMS-based interventions in enhancing antenatal care in developing countries: a systematic review
Source: BMJ Open. 2025 Feb 25;15(2):e089671. doi: 10.1136/bmjopen-2024-089671 (PMC11865757; doi:10.1136/bmjopen-2024-089671)
Supplement: online supplemental file 10 [file bmjopen-15-2-s010.pdf]

Supplementary tables

eTable 1 Full search strategy

	PubMed	Scopus	Web of Science	The Cochrane Library	**AISeL**	**Google Scholar**	**Science Direct**
Search done in	Title and Abstract	Title, Abstract and Keywords	Abstract	Title, Abstract and Keywords	All fields	All fields	All fields
Language	English					English and french	English
Year filter	2014-2024						
English search strings	("SMS-based applications" OR "text messaging" OR "mobile health" OR "mHealth") AND ("antenatal care" OR "prenatal care" OR "pregnancy care" OR "ANC") AND ("developing countries" OR "low-income countries" OR "resource-limited settings")						
French search string	("applications basées sur SMS" OU "messagerie texte" OU "santé mobile" OU "mSanté") ET ("soins prénatals" OU "soins anténataux" OU "soins pendant la grossesse" OU "CPN") ET ("pays en développement" OU "pays à faible revenu" OU "contextes à ressources limitées")						

eTable 2. Summary of search results

	PubMed	Scopus	Web of Science	The Cochrane Library	**AISeL**	**Google Scholar**		**Science Direct**	**Total**
						En	Fr		
**Results (considered)**	21	62	26	10	57	4750 (200)	256(200)	3207(200)	776
**Suitable studies**	3	1	0	0	1	5	0	2	12

eTable 3 Inclusion and exclusion criteria

Description	**Inclusion**	**Inclusion justification**	**Exclusion**	**Exclusion justification**
**Study Design**	Randomised controlled trials (RCTs), quasi-experimental, observational, and qualitative studies that provide data on the implementation,	Allows for a comprehensive understanding of different aspects of SMS-based interventions from efficacy to real-world	Editorials, opinion pieces, reviews, and studies without primary data or clear outcomes related to antenatal care and SMS-	They do not provide empirical data necessary for a systematic review

	usage, and outcomes of SMS-based antenatal care interventions.	application and user experiences	based interventions.	
**Population**	Studies involving pregnant women in developing countries. This can include women of all ages, ethnicities, and stages of pregnancy.	To ensure that the findings are relevant to populations where SMS-based interventions might be most necessary and effective due to limited healthcare access.	Studies focusing on populations outside of developing countries or on non-pregnant women.	To maintain the review's focus on the specific needs and context of pregnant women in resource-limited settings.
**Intervention**	Studies that focus on SMS-based applications designed to improve antenatal care information and attendance. That includes interventions promoting health education, appointment reminders, health monitoring, and support through text messaging.	To evaluate the effectiveness of this technology in enhancing ANC.	Studies that do not specifically use SMS-based communication as a primary method for delivering antenatal care information or support.	To ensure that the results are specific to the impact of SMS-based interventions without the confounding effects of other communication technologies.
**Comparators**	Studies with or without a control group. For those with a control group, the comparison can be standard care, no intervention, or other digital health interventions not using SMS.	To allow for a broader range of data on the effectiveness of SMS interventions, including comparative analyses against different forms of care.	Studies where the control group is subjected to interventions primarily based on SMS technology; studies that do not clearly describe the comparator.	To clearly distinguish the effect of SMS interventions from other variables.

**Outcomes**	Studies that measure outcomes related to antenatal care include improvements in antenatal care attendance, enhanced knowledge of antenatal health, improved pregnancy outcomes, and user satisfaction with the SMS service.	ensures that the review directly addresses the impact of SMS interventions on key health metrics and patient satisfaction.	Studies that do not report specific outcomes related to antenatal care	To maintain clarity and relevance.
**Publication date**	Studies published within the last ten years, from the year 2014	To ensure that the data reflects recent advancements in SMS technology and contemporary healthcare contexts.	Studies published more than ten years ago.	To avoid data that may not accurately reflect current technologies or healthcare practices.
**Language**	Content written in English or French	To expand the scope of the literature reviewed and due to language capabilities.	Content not written in English or French	To ensure quality due to language proficiency constraints.

eTable 4 Data extraction form

Item	Value
**Study Identification**	
Study ID	Identification
Author name	Name(s) of the author(s)
Title	Title of the paper
Journal	Journal where the paper is published
Year	Year of publication
Study design	randomised controlled trial, observational study, etc
Country	The country where the study was conducted
**Study Participants**	
Population description	Accurate description of the population
Sample size	value
Inclusion criteria	Main criteria reported

Exclusion criteria	Main criteria reported
**Intervention Details**	
Description of the SMS-based application	General description with purpose
Content of the SMS-based application	Content of the messages
Message sending frequency of the app	frequency of messages
Tools employed for the implementation of the app	Resources and tools employed for the implementation and/or requirements
Duration of the intervention	Duration as reported
Control or comparator interventions	As reported, if applicable
**Outcomes**	
Primary outcomes	Improve antenatal care attendance, knowledge enhancement, vaccination visits, satisfaction, etc.
Secondary outcomes	As reported, if applicable
Outcome measurement tools and methods	As reported
**Results**	
Key findings	Summary of results related to primary and secondary outcomes
Statistical significance	If applicable
Limitations	Limitations reported by the study
**Quality Assessment**	
Risk of bias assessment	For each study, depending on the study design

*eTable 5 Study design * Outcome*

		Outcome						
		Effectiveness				Safety		Others
		Primary		Secondary		Primary		
		Improved Attendance	Skilled delivery attendance	Increased Knowledge	patient satisfaction	Neonatal mortality	Reduced Complications	
Study design	RCT	7	2	1	1	1	0	1
	Non-RCT	2	2	1	0	0	1	1
	Other	0	0	1	0	0	0	1
Total		9	4	3	1	1	1	3

eTable 6. Impact of study design

Study Design	Total	Significant_Studies	Partial_Significant_Studies	Not Applicable_Studies	Regions covered
RCT	8	5	2	1	Ethiopia, Kenya, Nigeria, Pakistan, Tanzania, Uganda, Iraq
QE	3	3	0	0	Ghana, Tanzania, Timor-Leste
Sociotechnical approach using the prototype method.	1	1	0	0	Brazil
