# Supplementary material for: Effectiveness of SMS-based interventions in enhancing antenatal care in developing countries: a systematic review
Source: BMJ Open. 2025 Feb 25;15(2):e089671. doi: 10.1136/bmjopen-2024-089671 (PMC11865757; doi:10.1136/bmjopen-2024-089671)
Supplement: Supplementary file 11 [file bmjopen-2024-089671.reviewer_comments.pdf]

PEER REVIEW HISTORY

BMJ Open publishes all reviews undertaken for accepted manuscripts. Reviewers are asked to complete a checklist review form and are provided with free text boxes to elaborate on their assessment. These free text comments are reproduced below.

ARTICLE DETAILS

Title (Provisional)

Effectiveness of SMS-Based interventions in enhancing antenatal care in developing countries: systematic review

Authors

Kante, Mahamadou; Målqvist, Mats

VERSION 1 - REVIEW

Reviewer **1**

Name **Muthu Perumal, Prakash**

Affiliation **SRM Institute of Science and Technology, School of Public Health**

Date **27-Jul-2024**

COI interest **I understand and confirm that I don't have competing**

Enhancing Antenatal Care through SMS-Based Interventions in Developing Countries" provides a thorough analysis of the effectiveness of SMS interventions. However, it is important to note that SMS-based interventions, while impactful, may now seem somewhat outdated. With the advent of more advanced mHealth technologies such as voice messages, IVR systems, and social media platforms, there are new opportunities to enhance engagement and accessibility. Future reviews should consider these newer technologies to provide a more comprehensive understanding of current and potential mHealth interventions in antenatal care.

Reviewer **2**

Name **Moron-Duarte, Lina**

Affiliation **Universidade Federal de Pelotas Departmento de Medicina Social, Post-Graduate Program in Epidemiology**

Date **01-Aug-2024**

COI No

Title:

The title clearly identifies the study as a systematic review, which is positive.

Abstract

The abstract is clear and structured, covering objectives, methods, results, and conclusions.

Introduction

Rationale: The introduction adequately sets the context and importance of prenatal care in developing countries.

Objectives: The research questions (RQ1 and RQ2) are explicit and relevant. It is suggested that the authors include a brief discussion on the need for such interventions in the current public health context. Using the PICOT framework to structure the questions is recommended.

Methods

Eligibility Criteria: Inclusion and exclusion criteria are clearly defined, which is essential for transparency. It is recommended to provide more detail on the justification for these criteria.

Information Sources: Several databases are mentioned, indicating a thorough approach. However, it would be useful to include the date of the last search for each database.

Search Strategy: The search strategy is presented generally in the paper, but there are supplementary materials supporting each of the strategies used.

Selection Process: The document should detail the number of reviewers involved in the study selection and their independence. This is crucial for assessing the risk of bias in selection (this is described in the final part of the paper but should be stated in the methodology).

Data Collection: The methodology for data collection is adequate, but it is recommended to include information on how discrepancies between reviewers were handled, if any.

Results

Total Number of Studies Included: A summary of the number of studies and participants is provided, which is positive.

Results Synthesis: Results are presented clearly, but it would be beneficial to include a more detailed analysis of the heterogeneity among studies and how it was addressed in the synthesis.

Discussion

Limitations of the Evidence: Limitations of the included studies are discussed, which is fundamental. It is recommended that the authors delve deeper into the implications of these limitations for the interpretation of results.

Interpretation of Results: The discussion on the interpretation of results is adequate, but it is suggested to include practical recommendations for implementing these interventions in the context of prenatal care.

Others

Funding and Conflicts of Interest: The statement on funding and the absence of conflicts of interest is presented clearly, which is a positive aspect for the study's transparency.

Conclusions and Recommendations

The article presents a well-structured and relevant systematic review on SMS interventions in prenatal care. However, there are areas that could be improved to enhance the clarity and robustness of the study. The authors are encouraged to consider the following suggestions:

Expand the discussion on limitations and their implications for practice.

Include practical recommendations based on the findings to facilitate the implementation of SMS interventions in prenatal care.

Overall, the article has the potential to make a significant contribution to the field of public health and prenatal care, and with some improvements, it could serve as a valuable reference for researchers and practitioners in the area.

**Reviewer**	**3**
**Name**	**Bhaskar, Sonu**
**Affiliation**	**South Western Sydney Local Health District, Liverpool Hospital, Department of Neurology & Neurophysiology**
**Date**	**07-Nov-2024**
**COI**	**None**

The use of SMS-based interventions for antenatal care (ANC) is a promising area of study, and the systematic review sought a comprehensive understanding of existing literature and interventions. I have several major and minor concerns for authors to consider:

1. The manuscript acknowledges that data extraction and bias assessments were conducted by a single reviewer. This introduces a risk of bias and reduces the reliability of the findings. Multiple reviewers are crucial to ensure the objectivity and robustness of the review process.

2. The study did not perform a meta-analysis. This limits the ability to draw quantitative conclusions about the overall effectiveness of SMS-based interventions, vis a vis pooled effect sizes.

3. The studies included vary significantly in terms of their design, population, interventions, and outcomes measured. This heterogeneity makes it challenging to generalize the findings across different contexts.

4. A subgroup analysis based on study characteristics such as geographic location, type of SMS intervention, and specific outcomes measured is needed to identify which factors contribute most to the success of SMS-based interventions.

5. While the variability in intervention effectiveness is briefly discussed, suggest expanding why certain interventions may be more effective than others, including the role of message content, frequency, and cultural context.

VERSION 1 - AUTHOR RESPONSE

Reviewer: 1
Dr. Prakash Muthu Perumal, SRM Institute of Science and Technology
Comments to the Author:
Enhancing Antenatal Care through SMS-Based Interventions in Developing Countries" provides a thorough analysis of the effectiveness of SMS interventions. However, it is important to note that SMS-based interventions, while impactful, may now seem somewhat outdated. With the advent of more advanced mHealth technologies such as voice messages, IVR systems, and social media platforms, there are new opportunities to enhance engagement and accessibility. Future reviews should consider these newer technologies to provide a more comprehensive understanding of current and potential mHealth interventions in antenatal care.

Response:

Dear **Dr. Prakash Muthu Perumal**,

Thank you for your constructive comments and for indeed recognising the thorough analysis provided in our review of SMS-based interventions in antenatal care for developing countries. We appreciate your suggestion to consider more advanced mHealth technologies such as voice messages, IVR systems, and social media platforms.

We do agree that these technologies offer exciting possibilities for enhancing engagement and accessibility in maternal health initiatives. However, our review focused specifically on SMS-based interventions due to their continued relevance and applicability in the contexts of developing countries, where challenges such as **limited internet access, lower literacy rates, and the lack of basic necessities like consistent electricity and water supply prevail**. In these environments, the ubiquity and simplicity of SMS make it a uniquely powerful tool for reaching broad populations.

While we acknowledge the potential of newer technologies, our scope was intentionally focused to critically evaluate interventions feasible within the mentioned resource constraints. Nonetheless, your point is well-taken, and we agree that a future review could fruitfully expand to include these emerging

technologies, assessing their feasibility, effectiveness, and contextual appropriateness alongside traditional SMS-based approaches.

We will clarify this scope limitation in the revised version to ensure that readers understand the context-driven rationale behind our focus on SMS-based interventions.

Thank you

Reviewer 2:

Dr. Lina Moron-Duarte, Universidade Federal de Pelotas Departmento de Medicina Social
Comments to the Author:
Title:

The title clearly identifies the study as a systematic review, which is positive.

Response: thank you

Abstract

The abstract is clear and structured, covering objectives, methods, results, and conclusions.

Response: thank you

Introduction

Rationale: The introduction adequately sets the context and importance of prenatal care in developing countries.
Objectives: The research questions (RQ1 and RQ2) are explicit and relevant. It is suggested that the authors include a brief discussion on the need for such interventions in the current public health context. Using the PICOT framework to structure the questions is recommended.

Response:

We appreciate your suggestion to include a brief discussion on the need for SMS-based interventions in the current public health context and agree that incorporating the PICOT (Population, Intervention, Comparison, Outcome, Time) framework would enhance the structure and clarity of our research questions.

To address your recommendations, we expanded the introduction to edit a paragraph adding the need for accessible and effective maternal health interventions within the current global public health landscape. Additionally, we reframed our research questions using the PICOT format to provide a clearer and more structured approach. Please see the revised version.

Methods

Eligibility Criteria: Inclusion and exclusion criteria are clearly defined, which is essential for transparency. It is recommended to provide more detail on the justification for these criteria.

Response:

Thank you for acknowledging the clarity of our inclusion and exclusion criteria. Your suggestion to provide more detailed justification for these criteria is well taken.

We have included a table in the supplementary materials (eTable 3) that comprehensively details all the criteria used, along with **justifications** for each inclusion and exclusion criterion. Accordingly, we have slightly condensed their description within the main text. We believe this addition will enhance clarity regarding our selection process.

Information Sources: Several databases are mentioned, indicating a thorough approach. However, it would be useful to include the date of the last search for each database.

Response:

In accordance with this suggestion, we added the last search for each database. This was possible without much trouble because of the reference manager we use (Mendeley).

Search Strategy: The search strategy is presented generally in the paper, but there are supplementary materials supporting each of the strategies used.

Response: Indeed, thank you.

Selection Process: The document should detail the number of reviewers involved in the study selection and their independence. This is crucial for assessing the risk of bias in selection (this is described in the final part of the paper but should be stated in the methodology).

Response:

In agreement with this comment, in the methodology section, we specified that the study selection process was conducted by a single reviewer due to constraints in accessing independent reviewers with the necessary language proficiency and expertise in the subject matter. Financial limitations also prevented the recruitment of additional personnel. While this is a deviation from ideal systematic review practices, which recommend multiple independent reviewers to minimize bias, the circumstances of our study necessitated this approach. Full transparency regarding this aspect of our methodology is provided to allow for an accurate assessment of potential biases in the selection process.
Data Collection: The methodology for data collection is adequate, but it is recommended to include information on how discrepancies between reviewers were handled, if any.

Response: None (please refer to the previous response).

Results

Total Number of Studies Included: A summary of the number of studies and participants is provided, which is positive.

Response: Thank you
Results Synthesis: Results are presented clearly, but it would be beneficial to include a more detailed analysis of the heterogeneity among studies and how it was addressed in the synthesis.

Response:

Thank you for your constructive feedback on the presentation of our results. We appreciate the suggestion to enhance our analysis of heterogeneity among the included studies. In response, we included a more detailed discussion of the variations in study designs, populations, and outcomes in our results section. This involved subgroup analysis, to clarify how differences among studies influenced our conclusions. Tables and figures are given as supplementary files. This enhancement, we believe provide a proper insight into the reliability and applicability of our findings across varied contexts.

Given that this study is focused solely on a systematic review as mentioned in the title, we did not conduct quantitative statistical analyses typically required for meta-analysis, such as pooled effect size calculations or heterogeneity tests (e.g., prediction Intervals, or I^2 as most do (proportion of variance)) [1]. While these methods could have added quantitative depth, they were not necessary to achieve the primary objective of synthesising and qualitatively analysing the evidence to inform our research focus.

Discussion

Limitations of the Evidence: Limitations of the included studies are discussed, which is fundamental. It is recommended that the authors delve deeper into the implications of these limitations for the interpretation of results.

Response: In agreement with this comment, more discussion have been done regarding the limitations and their implications (please refer to the discussion section).
Interpretation of Results: The discussion on the interpretation of results is adequate, but it is suggested to include practical recommendations for implementing these interventions in the context of prenatal care.
Response: we have separated results from the discussion and practical recommendations have been made as well in the revised version (Section 4).

Others

Funding and Conflicts of Interest: The statement on funding and the absence of conflicts of interest is presented clearly, which is a positive aspect for the study's transparency.

Response: Thank you

Conclusions and Recommendations

The article presents a well-structured and relevant systematic review on SMS interventions in prenatal care. However, there are areas that could be improved to enhance the clarity and robustness of the study. The authors are encouraged to consider the following suggestions:

Expand the discussion on limitations and their implications for practice.
Include practical recommendations based on the findings to facilitate the implementation of SMS interventions in prenatal care.
Overall, the article has the potential to make a significant contribution to the field of public health

and prenatal care, and with some improvements, it could serve as a valuable reference for researchers and practitioners in the area.

Response:

Thank you for your overall constructive feedback on our systematic review. We appreciate your recognition of our efforts, the article's structure and relevance, as well as your insightful suggestions for enhancement. In response to your comments, we have broadened the discussion as indicated above on the study's limitations to provide a more comprehensive understanding of their implications for practice (please see section 4). Additionally, actionable recommendations that are grounded in our findings to support the effective implementation of SMS interventions in prenatal care settings have been added.

Reviewer: 3
Dr. Sonu Bhaskar, South Western Sydney Local Health District, Ingham Institute

Comments to the Author:
The use of SMS-based interventions for antenatal care (ANC) is a promising area of study, and the systematic review sought a comprehensive understanding of existing literature and interventions. I have several major and minor concerns for authors to consider:

1. The manuscript acknowledges that data extraction and bias assessments were conducted by a single reviewer. This introduces a risk of bias and reduces the reliability of the findings. Multiple reviewers are crucial to ensure the objectivity and robustness of the review process.

Response: Indeed, this is acknowledged in the paper. We have added more rationale for this limitation in the revise version. we have specified that the study selection process was conducted by a single reviewer due to constraints in accessing independent reviewers with the necessary language proficiency and expertise in the subject matter. Financial limitations also prevented the recruitment of additional personnel. While this is a deviation from ideal systematic review practices, which recommend multiple independent reviewers to minimize bias, the circumstances of our study necessitated this approach. Full transparency regarding this aspect of our methodology is provided to allow for an accurate assessment of potential biases in the selection process. Please see section 2.4.

2. The study did not perform a meta-analysis. This limits the ability to draw quantitative conclusions about the overall effectiveness of SMS-based interventions, vis a vis pooled effect sizes.

3. The studies included vary significantly in terms of their design, population, interventions, and outcomes measured. This heterogeneity makes it challenging to generalize the findings across different contexts.

Response to comment 2 and 3:

As specified, given that this study is focused solely on a systematic review as mentioned in the title, we did not conduct quantitative statistical analyses typically required for meta-analysis, such as pooled effect size calculations or heterogeneity tests (e.g. prediction Intervals, or I^2 as most do (proportion of variance)) [1]. While these methods could have added quantitative depth, they were not necessary to achieve the primary objective of synthesising and qualitatively analysing the evidence to inform our research focus.

4. A subgroup analysis based on study characteristics such as geographic location, type of SMS intervention, and specific outcomes measured is needed to identify which factors contribute most to the success of SMS-based interventions.

Response: In agreement with this suggestion, in response, we included a more detailed discussion of the variations in study designs, populations, and outcomes in our results section. This involved subgroup analysis, to clarify how differences among studies influenced our conclusions. Tables and figures are given as supplementary files. This enhancement, we believe provide a proper insight into the reliability and applicability of our findings across varied contexts.

5. While the variability in intervention effectiveness is briefly discussed, suggest expanding why certain interventions may be more effective than others, including the role of message content, frequency, and cultural context.

Response: Please, refer to the new discussion section. After we separated the results and discussion section, these points were addressed.

Final words:

Thank you for your efforts in helping us improve the overall quality of the paper.

Reference

1 Borenstein M. How to understand and report heterogeneity in a meta-analysis: The difference between I-squared and prediction intervals. *Integr Med Res*. 2023;12:101014. doi: 10.1016/j.imr.2023.101014

VERSION 2 - REVIEW

Reviewer **2**

Name **Moron-Duarte, Lina**

Affiliation **Universidade Federal de Pelotas Departmento de Medicina Social, Post-Graduate Program in Epidemiology**

Date **19-Jan-2025**

COI

Dear Authors,

I would like to express my gratitude for the opportunity to review your manuscript titled "Enhancing antenatal care through SMS-Based interventions in developing countries: A systematic review of applications and their effectiveness." This study addresses a relevant

and high-priority topic for improving maternal health outcomes in developing countries. I appreciate the effort invested in conducting this review and present below my observations and recommendations to strengthen your manuscript.

1. Title and Abstract

The title reflects the study's focus on effectiveness, but consider refining it for conciseness. For example: "Effectiveness of SMS-Based Interventions in Enhancing Antenatal Care in Developing Countries: systematic review"

In the abstract, include specific information about the methodological quality of the included studies and a summary of the main findings in quantitative terms. This will enhance clarity and utility for readers.

2. Introduction

The introduction adequately contextualizes the relevance of SMS-based interventions.

3. Methods

Currently, the outcomes are not clearly categorized in terms of effectiveness and safety, nor differentiated as primary and secondary. This lack of categorization may hinder the analysis, presentation, and interpretation of the results.

I suggest organizing the outcomes into two main categories:

• Effectiveness: For example, improvement in antenatal care information and attendance at prenatal appointments.

• Safety: For example, adverse effects related to SMS interventions or any outcomes related to maternal or fetal well-being.

Additionally, I recommend classifying the outcomes within these categories as primary (those most relevant to the study objectives) and secondary (additional or exploratory outcomes).

Although the search and selection process is described in detail, certain organizational issues are evident.

I recommend reorganizing this section to include exclusively:

• The complete search and selection process (with a mention of the PRISMA diagram).

• Inclusion/exclusion criteria.

• Tools used for quality assessment (e.g., RoB 2, ROBINS-I) and how they were applied.

• Clearly separate methods and results: Describe the selection stages in this section and focus solely on explaining the process, without including detailed figures, which should be presented in the results section.

• Explicitly declare whether the study was registered and, if so, provide the registration number. If the study was not registered, include it as a limitation and explain why this step was not carried out.

Although it is mentioned in the limitations that the entire process was carried out by a single reviewer, this information should be clearly described in the methods section.

I recommend:

• Explicitly state that screening, quality assessment, and data extraction were conducted by a single reviewer.

• Justify this methodology, mentioning potential constraints (e.g., resource limitations) and the tools used to minimize bias.

4.Results

I suggested restructuring the Results section into clear subsections:

• Search and selection results: Total number of studies found, excluded, and included.

• Methodological quality of studies: Summary of the risk of bias.

• Primary outcomes: Impact on information and antenatal attendance.

• Secondary outcomes: Perinatal mortality, satisfaction, etc.

5. Discussion

The discussion contextualizes the results well but does not sufficiently address how methodological quality affects the interpretation of findings.

Suggestion:

• Analyze the relationship between study quality and the robustness of the results.

• Reflect on how identified biases may have influenced the overall findings.

6. Limitations

While some limitations are mentioned, there is no discussion about the lack of detailed analysis of methodological quality in the results.

Suggestion: Explicitly highlight this omission as a limitation and propose how it could be addressed in future research.

General recommendation:

I recommend reviewing the writing to use shorter paragraphs or more concise ideas. Currently, the text is complex to read and simplifying it could enhance clarity and improve the reader’s understanding.

Thank you for your attention to these recommendations. I hope they are helpful in enhancing the quality and impact of your manuscript.

Lina Sofía Morón-Duarte

Reviewer 3

Name Bhaskar, Sonu

Affiliation South Western Sydney Local Health District, Liverpool Hospital, Department of Neurology & Neurophysiology

Date 12-Jan-2025

COI

The article systematically reviews SMS-based interventions for antenatal care (ANC) in low- and middle-income countries (LMICs), focusing on their applications and effectiveness. It highlights improvements in ANC attendance, maternal health knowledge, and service utilization through SMS interventions, particularly in East Africa. However, the study has several limitations, including reliance on a single reviewer, lack of meta-analytical statistical analysis, and underrepresentation of regions beyond East Africa.

1. Overemphasis on East Africa neglects other LMIC regions, which affects generalizability. Please expand.

2. The study lacks quantitative meta-analyses, which limits the depth of statistical conclusions.

3. In the introduction, please include recent references to strengthen claims about the utility of SMS-based interventions. Clarify how this review builds upon existing systematic reviews.

4. Methods: The inclusion of only English and French studies creates language bias, excluding relevant research. While tools like RoB 2 and ROBINS-I are appropriate, the process is not independently validated.

5. Provide effect sizes and confidence intervals for all included studies for consistency. Add a forest plot summarizing outcomes to improve clarity.

6. There is overemphasis on East Africa without sufficient exploration of underrepresented regions. There is also. a lack of of critical discussion on why some interventions fail.

VERSION 2 - AUTHOR RESPONSE

Reviewer: 3
Dr. Sonu Bhaskar, South Western Sydney Local Health District, Ingham Institute
Comments to the Author:
The article systematically reviews SMS-based interventions for antenatal care (ANC) in low- and middle-income countries (LMICs), focusing on their applications and effectiveness. It highlights improvements in ANC attendance, maternal health knowledge, and service utilization through SMS

interventions, particularly in East Africa. However, the study has several limitations, including reliance on a single reviewer, lack of meta-analytical statistical analysis, and underrepresentation of regions beyond East Africa.

1. Overemphasis on East Africa neglects other LMIC regions, which affects generalizability. Please expand.

Response:

We appreciate this comment and the opportunity to clarify the geographical representation in our review. Our findings reflect the results of a systematic search based on **predefined procedures and a rigorous research question**. As such, we included all relevant studies identified through our search strategy. While there is a concentration of studies from East Africa, we also reported findings from other regions, including **Ghana, Nigeria, Iraq, Pakistan, Timor-Leste, and Brazil**.

It is worth noting that the apparent overrepresentation of East African studies may be due to the higher number of SMS-based interventions conducted and published in that region (5 among the 12 studies). If evidence from other LMICs exists, it was not captured through our systematic search. We have highlighted this limitation in the discussion section (page 17 and 18) to underscore that our findings are reflective of the existing literature, rather than an **intentional focus** on any specific region.

2. The study lacks quantitative meta-analyses, which limits the depth of statistical conclusions.

Response:

Given that this study is focused solely on a systematic review as mentioned in the title, we did not conduct quantitative statistical analyses typically required for meta-analysis, such as pooled effect size calculations or heterogeneity tests (e.g., prediction Intervals, or I^2 as most do (proportion of variance)) [1]. While these methods could have added quantitative depth, they were not necessary to achieve the primary objective of synthesising and qualitatively analysing the evidence to inform our research focus.

3. In the introduction, please include recent references to strengthen claims about the utility of SMS-based interventions. Clarify how this review builds upon existing systematic reviews.

Response

In accordance with this comment, we have added recent references. Please refer to the introduction (page 2-3).

4. Methods: The inclusion of only English and French studies creates language bias, excluding relevant research. While tools like RoB 2 and ROBINS-I are appropriate, the process is not independently validated.

Response:

We appreciate the reviewer's observation regarding the potential for language bias. While we acknowledge that limiting our review to studies published in English and French may exclude relevant research in other languages, we also believe that considering two widely spoken languages represent an improvement over reviews that focus solely on **English**. This approach was a logical choice, as it

aligns with our linguistic expertise, ensuring accurate interpretation and critical appraisal of the included studies.

Regarding the use of RoB 2 and ROBINS-I, we carefully followed established guidelines for their application to ensure rigor and consistency. While independent validation of the risk of bias assessment is ideal, as mentioned in the paper, resource constraints did not allow for this in the current study.

We hope this addresses the reviewer’s concerns and provides sufficient clarification.

5. Provide effect sizes and confidence intervals for all included studies for consistency. Add a forest plot summarizing outcomes to improve clarity.

Response:

Given that this study is focused solely on a systematic review as mentioned in the title, we did not conduct quantitative statistical analyses typically required for meta-analysis, such as pooled effect size calculations or heterogeneity tests (e.g., prediction Intervals, or I^2 as most do (proportion of variance)) [1]. While these methods could have added quantitative depth, they were not necessary to achieve the primary objective of synthesising and qualitatively analysing the evidence to inform our research focus.
6. There is overemphasis on East Africa without sufficient exploration of underrepresented regions. There is also. A lack of critical discussion on why some interventions fails.

Response:

Please refer to our response to comment 1.

Reviewer: 2

Dr. Lina Moron-Duarte, Universidade Federal de Pelotas Departmento de Medicina Social
Comments to the Author:

Dear Authors,
I would like to express my gratitude for the opportunity to review your manuscript titled “Enhancing antenatal care through SMS-Based interventions in developing countries: A systematic review of applications and their effectiveness.” This study addresses a relevant and high-priority topic for improving maternal health outcomes in developing countries. I appreciate the effort invested in conducting this review and present below my observations and recommendations to strengthen your manuscript.

1. Title and Abstract
The title reflects the study’s focus on effectiveness, but consider refining it for conciseness. For example: “Effectiveness of SMS-Based Interventions in Enhancing Antenatal Care in Developing Countries: systematic review”

Response:

In accordance with this suggestion, we agree to change the title for conciseness.

In the abstract, include specific information about the methodological quality of the included studies

and a summary of the main findings in quantitative terms. This will enhance clarity and utility for readers.

Response: Ok
2. Introduction
The introduction adequately contextualizes the relevance of SMS-based interventions.

Response: Thank you
3. Methods
Currently, the outcomes are not clearly categorized in terms of effectiveness and safety, nor differentiated as primary and secondary. This lack of categorization may hinder the analysis, presentation, and interpretation of the results.
I suggest organizing the outcomes into two main categories:
• Effectiveness: For example, improvement in antenatal care information and attendance at prenatal appointments.
• Safety: For example, adverse effects related to SMS interventions or any outcomes related to maternal or foetal well-being.
Additionally, I recommend classifying the outcomes within these categories as primary (those most relevant to the study objectives) and secondary (additional or exploratory outcomes).

Response: We appreciate the reviewer's suggestion to categorise the outcomes into effectiveness and safety and further distinguish them as primary and secondary outcomes. We have now implemented this classification in the results section (*see page 13 and eTable 5*), which we believe improves clarity for the readers.

In addition to this categorisation, we have retained our initial approach, which presents the evidence alongside the characteristics of the SMS-based interventions and subgroup analysis. We believe that maintaining this structure enhances the contextual understanding of the findings, ensuring that both the outcomes and the specific features of the interventions remain clear to the reader.

We hope this revision meets the reviewer's expectations while preserving the comprehensiveness of our initial analytical framework. Please let us know if further refinements are needed.

Comment
Although the search and selection process is described in detail, certain organizational issues are evident.
I recommend reorganizing this section to include exclusively:
• The complete search and selection process (with a mention of the PRISMA diagram).
• Inclusion/exclusion criteria.
• Tools used for quality assessment (e.g., RoB 2, ROBINS-I) and how they were applied.
• Clearly separate methods and results: Describe the selection stages in this section and focus solely on explaining the process, without including detailed figures, which should be presented in the results section.

Response:

We appreciate the reviewer's suggestions regarding the organisation of the methodology section. However, we respectfully maintain that the selection stages and the number of papers selected are integral parts of the methodology, as they directly pertain to the systematic search and selection process rather than the study's research findings. In our understanding, search results (i.e., the number of studies identified, screened, and included) form part of the methodological rigor and transparency of a systematic review, rather than the "results" of our research questions.

Furthermore, we have already provided a detailed description of the search process, inclusion/exclusion criteria, and the tools used for quality assessment (RoB 2, ROBINS-I), along with how they were applied. For clarity, we have carefully reviewed this section to ensure that it is well-structured and adheres to reporting standards.

If further reorganisation is required, we would appreciate specific guidance on which aspects need improvement while ensuring that the methodological integrity of the review remains intact. We hope this response clarifies our rationale.

Comment
• Explicitly declare whether the study was registered and, if so, provide the registration number. If the study was not registered, include it as a limitation and explain why this step was not carried out.

Response:

We appreciate the reviewer's suggestion regarding study registration. While prospective registration is increasingly encouraged for systematic reviews, it is not a mandatory requirement, particularly when the review does not involve meta-analysis or clinical trial data. In this case, our study followed a rigorous and transparent systematic review methodology, including a clearly defined search strategy, inclusion/exclusion criteria, and risk of bias assessment, ensuring methodological integrity.

Since this review was not pre-registered in databases such as PROSPERO, we acknowledge this as a limitation and have added a statement (page 18). However, we emphasise that all necessary methodological details are comprehensively reported to ensure reproducibility and transparency.

We hope this clarification adequately addresses the reviewer's concern.

Reviewer comment:
Although it is mentioned in the limitations that the entire process was carried out by a single reviewer, this information should be clearly described in the methods section.
I recommend:
• Explicitly state that screening, quality assessment, and data extraction were conducted by a single reviewer.
• Justify this methodology, mentioning potential constraints (e.g., resource limitations) and the tools used to minimize bias.

Response:

We appreciate the reviewer's recommendation regarding the description of the review process. **In our previous revision, we moved this information to the limitations section following a suggestion from a prior reviewer.** However, we recognise the importance of explicitly stating this in the methodology as well.

To address this concern, we have reintroduced a clear statement in the methods section **right in the first paragraph of the section before sub-sections** (see page 3) specifying that screening, quality assessment, and data extraction were conducted by a single reviewer. Additionally, we have provided a justification, citing resource limitations, language limitations, and the steps taken to minimise potential bias, including adherence to standardised tools such as RoB 2 and ROBINS-I.

We note that this information is already mentioned in the Strengths and Limitations section at the beginning of the paper. However, to ensure clarity, we have now expanded on it in the methods section while keeping it acknowledged in the limitations.

We hope this revision sufficiently addresses the reviewer's concern.

4.Results
I suggested restructuring the Results section into clear subsections:
• Search and selection results: Total number of studies found, excluded, and included.
• Methodological quality of studies: Summary of the risk of bias.

Response: please see our previous response about this in 3.
• Primary outcomes: Impact on information and antenatal attendance.
• Secondary outcomes: Perinatal mortality, satisfaction, etc.

Response: please see our previous response
5. Discussion
The discussion contextualizes the results well but does not sufficiently address how methodological quality affects the interpretation of findings.
Suggestion:
• Analyze the relationship between study quality and the robustness of the results.
• Reflect on how identified biases may have influenced the overall findings.

Response: In agreement with these suggestions, we made changes. Please refer to the discussion section (from page 17).
6. Limitations
While some limitations are mentioned, there is no discussion about the lack of detailed analysis of methodological quality in the results.
Suggestion: Explicitly highlight this omission as a limitation and propose how it could be addressed in future research.

Response: methodological biases were measured, reported and discussed.
General recommendation:
I recommend reviewing the writing to use shorter paragraphs or more concise ideas. Currently, the text is complex to read and simplifying it could enhance clarity and improve the reader's understanding.

Thank you for your attention to these recommendations. I hope they are helpful in enhancing the quality and impact of your manuscript.

Response:

We appreciate the reviewer's suggestion to improve readability by using shorter paragraphs and more concise phrasing. To address this, we have reviewed the manuscript and made some adjustments:

- Refined lengthy sentences to improve clarity and ensure smoother reading.
- Eliminated redundant wording while maintaining the scientific rigor and accuracy of the content.

Final words:

Thank you for your efforts in helping us improve the overall quality of the paper.

Reference

1 Borenstein M. How to understand and report heterogeneity in a meta-analysis: The

difference between I-squared and prediction intervals. *Integr Med Res*. 2023;12:101014. doi: 10.1016/j.imr.2023.101014
